# Continuous-flow synthesis of highly functionalized imidazo-oxadiazoles facilitated by microfluidic extraction

**DOI:** 10.3762/bjoc.13.26

**Published:** 2017-02-07

**Authors:** Ananda Herath, Nicholas D P Cosford

**Affiliations:** 1 Cancer Metabolism & Signaling Networks Program, Sanford Burnham Prebys Medical Discovery Institute, 10901 North Torrey Pines Road, La Jolla, California 92037, USA

**Keywords:** imidazo[1,2-*a*]pyridine, imidazo[1,2-*a*]pyridin-2-yl-1,2,4-oxadiazole, liquid–liquid extraction module, microreactor, 1,2,4-oxadiazole

## Abstract

A versatile continuous-flow synthesis of highly functionalized 1,2,4-oxadiazoles starting from carboxylic acids is reported. This process was applied to the multistep synthesis of imidazo[1,2-*a*]pyridin-2-yl-1,2,4-oxadiazoles, using a three reactor, multistep continuous-flow system without isolation of intermediates. This continuous-flow method was successfully combined with a single-step liquid–liquid microextraction unit to remove high boiling point polar solvents and impurities and provides the target compounds in high purity with excellent overall yields.

## Introduction

The design and execution of scalable and economically viable processes for the preparation of biologically active small molecules is a major hurdle in modern organic synthesis. The development of batch processes that combine multiple reactions into “one-pot” have been used successfully in some cases [1–6]. However, this approach has a number of drawbacks, primarily because of mutual interference between various reactive components. Recently, continuous-flow chemistry has emerged as a powerful technique in organic synthesis. This is in part due to the potential for integrating individual reaction steps and subsequent separations into a single streamlined process [7–14].

On the other hand, a significant challenge in flow chemistry is the formation of insoluble intermediates in the reactor. This can often be prevented by using polar organic solvents such as dimethylformamide (DMF) [7–9,15]. However, the challenges of removing large amounts of high boiling solvents during the purification and isolation process can limit scalability and efficiency. Furthermore, many useful synthetic reactions are incompatible with the use of DMF as a solvent. Recently several unit operations have successfully been implemented in continuous-flow syntheses to allow separations and purifications in a continuous fashion, such as liquid–liquid microextraction [12,16–20] or microfluidic distillation [21–22]. Herein we describe the utilization of liquid–liquid microextraction to facilitate a complex, multistep flow synthesis process.

Our research in the field of flow synthesis has focused on developing continuous-flow chemistry methods to access complex, drug-like molecules from readily available precursors without isolation of intermediates. We have shown that the “telescoping” of multiple synthetic steps into a single continuous process provides an efficient method for the production of heterocyclic compound libraries in sufficient quantities for biological screening in high-throughput assay formats as well as follow-up confirmatory studies. We previously reported a method for the preparation of 1,2,4-oxadiazoles in an uninterrupted continuous-flow sequence using arylnitriles and acyl chloride precursors [9]. We also reported the flow synthesis of highly functionalized imidazo[1,2-*a*]heteroaryl derivatives from readily available starting materials in a single continuous process [7]. We now report an efficient continuous-flow procedure for the synthesis of 1,2,4-oxadiazoles directly from arylnitriles and carboxylic acid derivatives. We further demonstrate the incorporation of this procedure into a continuous, three-microreactor method for the highly efficient preparation of a diverse library of imidazo-oxadiazole derivatives. Moreover, this continuous-flow method was successfully combined with a single-step liquid–liquid microextraction unit to remove high boiling point polar solvents and impurities.

## Results and Discussion

**Scheme 1:**
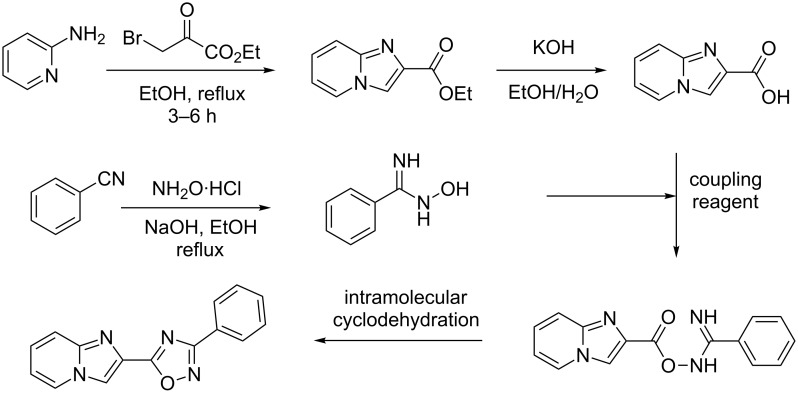
In-flask (batch) preparation of imidazo[1,2-*a*]pyridin-2-yl-1,2,4-oxadiazoles (S1P_1_ agonists) [27].

Historically the 1,2,4-oxadiazole scaffold has been used by medicinal chemists as a ubiquitous bioisosteric replacement of amide and ester functionalities in a wide variety of biologically active compounds [23–24]. This motif is found in several drugs and drug leads including sphingosine-1-phosphate 1 (S1P_1_) receptor agonists [25–27] and metabotropic glutamate subtype 5 (mGlu_5_) receptor negative allosteric modulators (NAMs) [25,28–30]. Most synthetic efforts toward the preparation of these heterocyclic systems utilize a multistep, in-flask approach as illustrated by the synthesis of S1P_1_ agonists (Scheme 1). Thus, a typical batch synthesis entails the formation of an amidoxime by reacting an arylnitrile with hydroxylamine in the presence of a base [29–31]. The amidoxime is then combined with a carboxylic acid derivative in the presence of a coupling reagent. The target oxadiazole is then formed via an intramolecular cyclodehydration (Scheme 1) [27,32–34].

For our initial studies on the development of a flow synthesis of 1,2,4-oxadiazoles we focused on the reaction of *N*-hydroxynicotinimidamide with 3-bromobenzoic acid (Table 1). Screening a variety of reaction conditions using a single microreactor we found that the combination of EDC/HOBt/DIPEA (1:1:1) for 10 min at 150 °C provided the best conditions for complete conversion of 3-bromobenzoic acid to the corresponding 1,2,4-oxadiazole derivative (Table 1, entry 5). The use of *N*,*N*-dimethylformamide (DMF) as a solvent resulted in competitive amide formation from the decomposition product of DMF at high temperature. In order to prevent this we switched to *N*,*N*-dimethylacetamide (DMA) as the solvent.

**Table 1:** Optimization of the flow synthesis of 1,2,4-oxadiazoles.

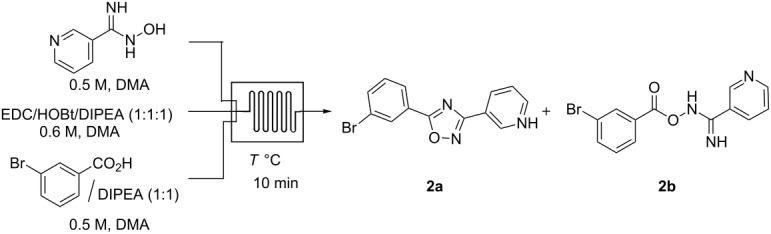		

Entry	*T* [°C]	Product Ratio **2a**:**2b**^a^

1	50	0:100^b^
2	75	3:97
3	100	18:82
4	125	80:20
5	150	99:1

^a^Compound ratios were determined using LC–MS (see Figure S1 in Supporting Information File 1). ^b^Compound **2a** was not observed.

We next focused our efforts on combining this optimized oxadiazole ring-closure procedure with our previously reported flow synthesis of amidoximes by the reaction of hydroxylamine with precursor aryl- and heteroarylnitriles (Table 2). These two reactions were successfully perfomed in flow with slight modifications to the reaction conditions to generate a variety of 1,2,4-oxadiazoles (Table 2). Reactions of arylnitriles having electron-donating (Table 2, entry 7) or electron-withdrawing groups (Table 2, entries 1, 2, 4 and 8) proceeded efficiently in good to excellent overall yields. Additionally, a range of aliphatic and aromatic acids were tolerated under these reaction conditions to produce the corresponding oxadiazoles in high yields. Several advantages of this methodology compared to our previously reported flow synthesis should be noted. First, this method is more facile because no cooling step is necessary before flowing into the second microreactor. Secondly, many more carboxylic acid derivatives are readily available (purchased or easily synthesized) than acyl chlorides allowing access to greater diversity. Finally, this method is easily adapatable to the synthesis of compounds with increasing complexity, as shown by our next set of experiments.

**Table 2:** Synthesis of 1,2,4-oxadiazoles via a continuous microreactor sequence from arylnitriles and carboxylic acids.

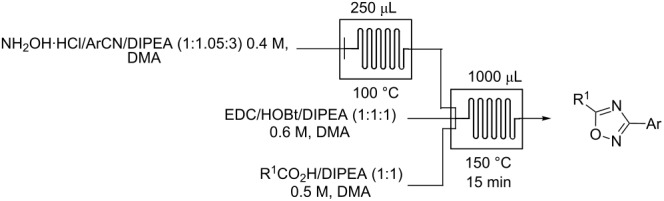					

Entry	Compound	Yield (%)^a^	Entry	Compound	Yield (%)^a^

1	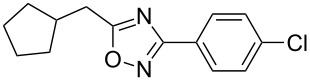	81	5	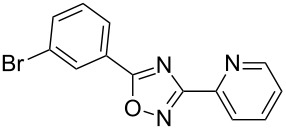	69
2	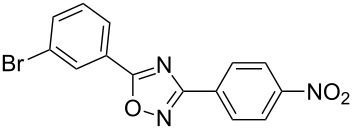	53	6	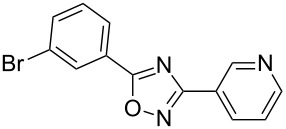	80
3	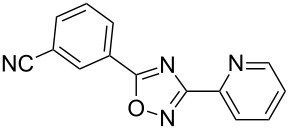	75	7	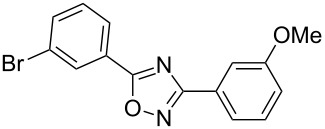	78
4	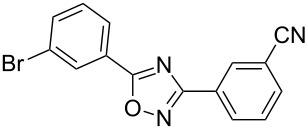	88	8	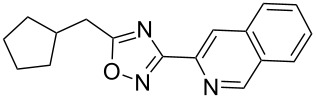	69

^a^Isolated yield after chromatographic purification of the crude reaction mixture.

To demonstrate the utility of the newly developed methodology, our next goal was to incorporate a carboxylic acid synthesis step into the flow process. As noted above, we previously reported the continuous-flow synthesis of imidazo[1,2-*a*]pyridine-2-carboxylic acids in a single, uninterrupted process directly from readily available starting materials. We hypothesized that incorporating this step into our new oxadiazole synthesis would provide access to diverse imidazo[1,2-*a*]pyridin-2-yl-1,2,4-oxadiazoles of biological importance [27]. The flow platform we were using, the Syrris AFRICA^®^ flow system, is limited to two heated reactors. To overcome this issue a third reactor was placed in a heated silicone oil bath and a flow system was assembled as shown in Table 3. The first reaction, the formation of imidazo[1,2-*a*]pyridine-2-carboxylic acid, was performed in a 1000 μL reactor (glass chip) at 100 °C. The acid exiting the first reactor was combined with EDC/HOBt/DIPEA (1:1:1) in a T-mixer. The synthesis of amidoxime was achieved by placing a second reactor (250 μL glass chip) in a heated silicone oil bath at 100 ^o^C. The product stream was next introduced into a third reactor (1000 μL) and mixed with the stream exiting from the T-mixer at 150 ^o^C. Initial studies suggested that premixing of acid and coupling reagent was efficient and provided better yields. The substrate scope of this continuous flow method is shown in Table 3. Thus, this flow method delivers a diverse array of drug-like heterocycles in good overall yields.

**Table 3:** Continuous-flow process for the synthesis of imidazo[1,2-*a*]pyridin-2-yl-1,2,4-oxadiazoles.

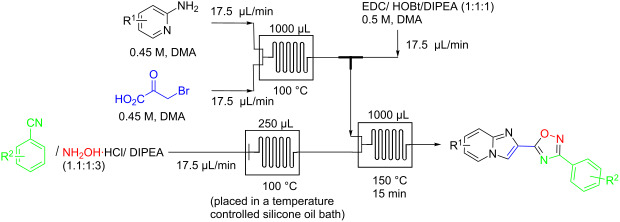					

Entry	Compound	Yield (%)^a^	Entry	Compound	Yield (%)^a^

1	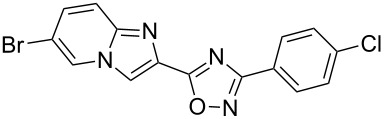	46	7	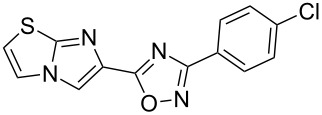	59
2	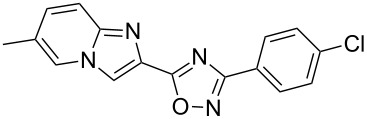	32	8	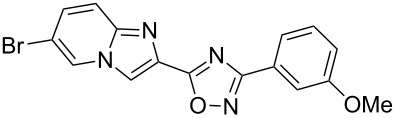	35
3	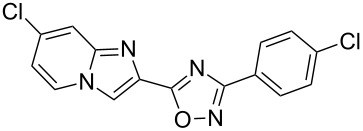	44	9	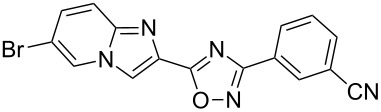	35
4	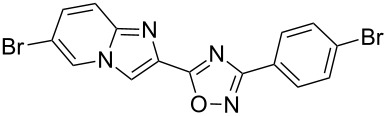	42	10	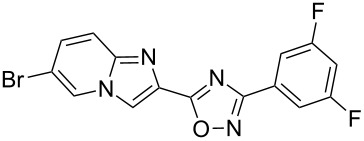	29
5	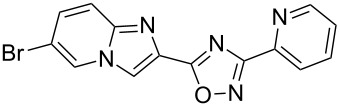	55	11	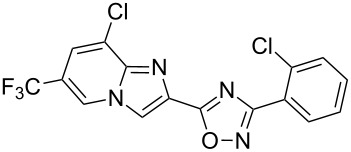	20
6	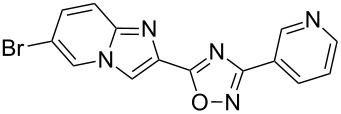	59	12	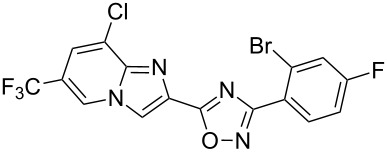	25

^a^Isolated yield after chromatographic purification of the crude reaction mixture.

**Scheme 2:**
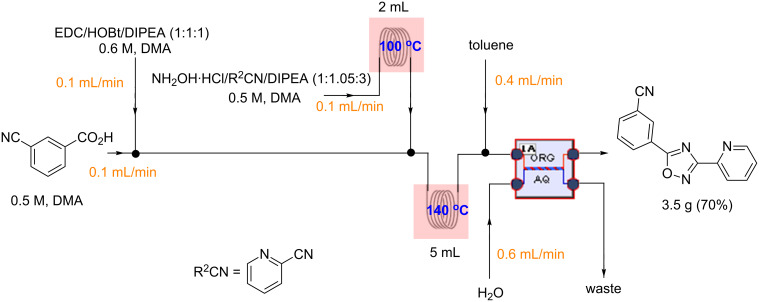
Gram-scale synthesis of mGlu_5_ NAM by continuous flow in combination with microfluidic extraction.

**Scheme 3:**
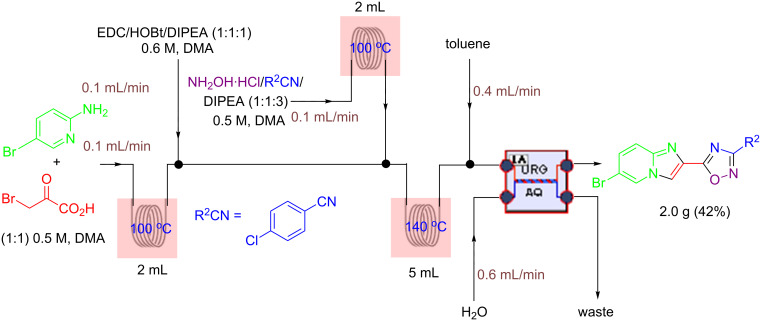
Gram-scale synthesis of imidazo[1,2-*a*]pyridin-2-yl-1,2,4-oxadiazole S1P_1_ agonist scaffold by continuous flow combined with microfluidic extraction.

Although this multistep continuous-flow method allowed the construction of complex molecules rapidly without the need for purification and isolation of intermediates, lower flow rates and small reactors limited the scalability of the method. As noted previously, the use of high boiling point solvents, such as DMA, are less attractive during scale-up due to the difficulty of removal of these solvents during purification. Consequently the current method is limited to small scale library synthesis. Therefore, our next goal was to convert this microfluidic procedure to a sequence that would deliver compounds on a larger scale. To this end, we first focused our attention on increasing the throughput of the reaction by using the larger tubing and tube reactors available in the Vapourtec R series flow system. We also increased the flow rate to maintain a residence time of 12 min in the third reactor (Scheme 2). Next, a microfluidic liquid–liquid extraction module (the AFRICA^®^ FLLEX) was incorporated at the end of the flow sequence to remove the high boiling point solvent (DMA). Initial studies using a single microreactor and dichloromethane as the organic extraction phase demonstrated proof-of-concept that the microfluidic extraction could be used in the oxadiazole flow synthesis procedure (see Supporting Information File 1). However, the optimized method employs the introduction of toluene and water into the microfluidic extraction module to efficiently remove the DMA and avoid the use of a halogenated solvent, as shown in Scheme 2 and Scheme 3. The reaction mixture exiting from the third reactor is mixed with water and toluene using an external pump before entering the phase separation device. On a preparative scale using this flow reaction setup, the desired oxadiazole derivatives were obtained in good yield with a throughput of ~0.5 g/h. To demonstrate the utility of this new method we synthesized the mGlu_5_ NAM (Table 2, entry 3) on a gram scale in high yield (3.5 g, 70%). With the same optimized flow method we also synthesized 5-(6-bromoimidazo[1,2-*a*]pyridin-2-yl)-3-(4-chlorophenyl)-1,2,4-oxadiazole on a gram scale (2 g, 42%) rapidly and efficiently (Scheme 3).

Several advantages of this methodology compared to the standard batch synthesis should be noted. First, a fully automated flow method permits the rapid construction of libraries of highly functionalized oxadiazole derivatives. Second, imidazo[1,2-*a*]pyridin-2-yl-1,2,4-oxadiazoles, a scaffold with activity as S1P_1_ agonists, are prepared directly from commercially available building blocks in a single continuous process without isolating intermediates. As noted previously, the standard in-flask (batch) synthesis of these compounds involves multiple reaction steps requiring work-up and purification of several intermediates. Furthermore, liquid–liquid microextraction removes high boiling point solvent and impurities from the product. Finally, this new process with liquid–liquid extraction allows easy scale-up and eliminates the tedious high boiling point solvent removal step.

## Conclusion

In summary, we have developed a telescoped continuous-flow method for the synthesis of diversely substituted imidazo[1,2-*a*]pyridine-2-yl-1,2,4-oxadiazole derivatives directly from commercial nitriles, aminopyridine carboxylic acids and hydroxylamine. Moreover, we demonstrated that a liquid–liquid microextraction unit can be utilized to remove high polar water soluble solvents and impurities from products. This scalable method provides the desired oxadiazole derivatives at a rate of ≈0.5 g/h and represents a significant advantage over batch synthesis. We anticipate that these advances will facilitate the rapid synthesis of these biologically important compounds.

## Experimental

All reagents were used as received unless otherwise noted. ^1^H and ^13^C spectra were obtained in CDCl_3_ at room temperature, unless otherwise noted, on a JEOL (JNM-CS400) 400 MHz instrument. Chemical shifts of ^1^H NMR spectra were recorded in parts per million (ppm) on the δ scale from an internal standard of residual CDCl_3_ (7.24 ppm). Chemical shifts of ^13^C NMR spectra were recorded in ppm from the central peak of CDCl_3_ (77.0 ppm) on the δ scale. High-resolution ESI–TOF mass spectra were acquired from the Mass Spectrometry Core at the Sanford Burnham Prebys Medical Discovery Institute (Orlando, Florida). LC–MS analyses were carried out on a Shimadzu LC–MS 2010 Series LC System with a Kromasil 100 5 micron C18 column (50 × 2.1 mm i.d.). Continuous-flow (microreactor) experiments were carried out using a Syrris AFRICA apparatus or a Vapourtec R Series Flow Chemistry System.

### General procedure for the optimization of the flow synthesis of 1,2,4-oxadiazoles (Table 1)

The reaction was conducted in a glass reactor consisting of a 1.0 mL retention unit and three inlets. Streams of EDC/HOBt/DIPEA (1:1:1, 25 μL/min, 0.6 M, DMA), acid/DIPEA (1:1, 25 μL/min, 0.5 M, DMA) and a solution of amidoximes (25 μL/min, 0.5 M, DMA) were combined in the glass reactor at different temperatures for 10 min of residence time. Reactions were monitored by LC–MS analysis and showed that the conversion of the 3-bromobenzoic acid to the corresponding 1,2,4-oxadiazole was optimal at 150 °C (Table 1, entry 5).

### General procedure for the synthesis of 1,2,4-oxadiazoles via a continuous microreactor sequence from arylnitriles and acids (Table 2)

A solution of ArCN/NH_2_OH·HCl/DIPEA (1.05:1:3, 0.4 M, DMA) was introduced to a glass microreactor (250 μL) heated at 100 °C. The stream exiting from the first reactor was combined with streams of the acid/DIPEA (1:1, 25.0 μL/min, 0.5 M, DMA) and EDC/HOBt/DIPEA (1:1:1, 25 μL/min, 0.6 M, DMA) in a second glass reactor (1.0 mL) at 150 °C for 15 min of residence time. This reaction was carried out with a back pressure of 4.0 bar. The reaction mixture was mixed with excess water and extracted three times with dichloromethane. The combined organic layers were washed with brine, dried over magnesium sulfate, filtered, and concentrated and the residue was purified via automated flash chromatography (SiO_2_) to afford the desired product (CombiFlash^®^ Rf, 12 g flash column). The solvent gradient was 90% hexane to 50% ethyl acetate over 15 min at a flow rate of 15 mL/min.

### General procedure for the continuous flow synthesis of imidazo[1,2-*a*]pyridin-2-yl-1,2,4-oxadiazoles (Table 3)

The first reaction, the formation of imidazo[1,2-*a*]pyridine-2-carboxylic acid, was carried out in a 1000 μL reactor (glass chip) at 100 °C. The acid exiting the first reactor was combined with EDC/HOBt/DIPEA (1:1:1, 0.5 M, DMA) in a T-mixer. The synthesis of amidoxime (ArCN/NH_2_OH·HCl/DIPEA (1.1:1:3), 0.4 M, DMA) was achieved by placing a second reactor (250 μL glass chip) in a heated silicone oil bath at 100 °C. This stream was next introduced into a third reactor and mixed with the stream exiting from the T-mixer at 150 °C. The stream exiting the third chip was collected after passing through the back pressure regulator. This reaction was carried out with a back pressure of 4.0 bar. The reaction mixture was mixed with excess water and extracted three times with dichloromethane. The combined organic layers were washed with brine, dried over magnesium sulfate, filtered, and concentrated and the residue was purified via automated flash chromatography (SiO_2_) to afford the desired product (CombiFlash^®^ Rf, 12g flash column). The solvent gradient was 90% hexane to 50% ethyl acetate over 15 min at a flow rate of 15 mL/min.

## Supporting Information

LC traces for optimization of oxadiazole synthesis (Table 1), details of liquid–liquid microextraction with FLLEX module including LC traces, general and detailed synthetic procedures with full characterization data for compounds, and ^1^H NMR and ^13^C NMR spectral traces of all compounds.

File 1 Experimental data.
