# Supplementary material for: Continuous-flow synthesis of highly functionalized imidazo-oxadiazoles facilitated by microfluidic extraction
Source: Beilstein J Org Chem. 2017 Feb 7;13:239–46. doi: 10.3762/bjoc.13.26 (PMC5331298; doi:10.3762/bjoc.13.26)
Supplement: File 1 — Experimental data. [file Beilstein_J_Org_Chem-13-239-s001.pdf]

Supporting Information

for

Continuous-flow synthesis of highly functionalized imidazo-oxadiazoles facilitated by microfluidic extraction

Ananda Herath and Nicholas D. P. Cosford*

Address: Cancer Metabolism & Signaling Networks Program, Sanford Burnham Prebys Medical Discovery Institute, 10901 North Torrey Pines Road, La Jolla, California 92037, USA

Email: Nicholas D. P. Cosford - ncosford@sbpdiscovery.org

*Corresponding author

Experimental data

Entry 1, T = 50 ^{o}C

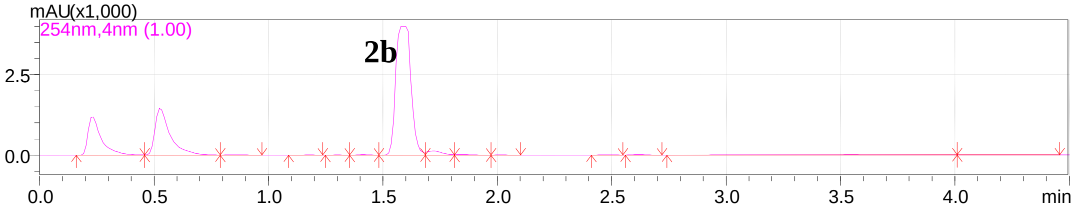

Entry 2, T = 75 ^{o}C

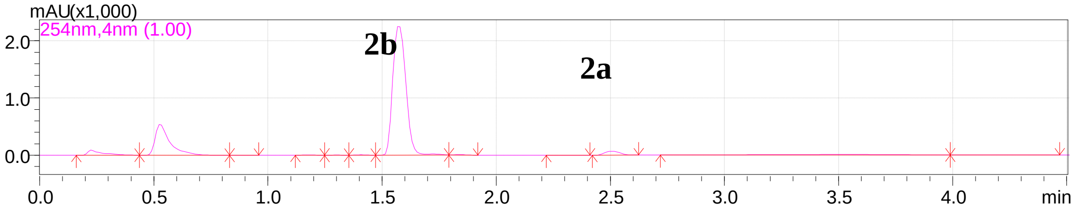

Entry 3, T = 100 ^{o}C

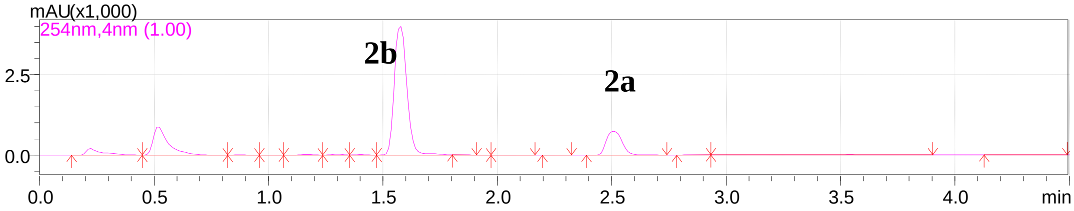

Entry 4, T = 125 ^{o}C

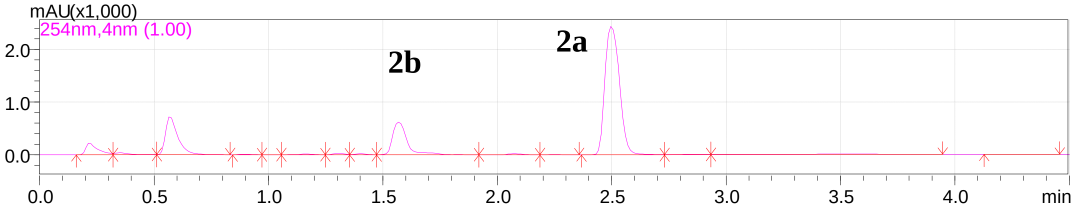

Entry 5, T = 150 ^{o}C

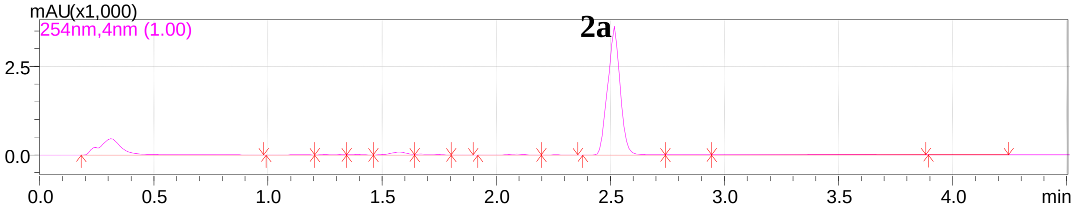

Figure S1: LC–MS traces used to determine compound ratios for experiments in Table 1.

Optimization of liquid–liquid microfluidic extraction

The AFRICA® fluidic liquid-liquid extraction (FLLEX) module consists of a contactor, a separator, two optical sensors to monitor output stream purity and two back pressure regulators to accurately control fluid Pressure (Figure S2). Separation is achieved by passing the slug flow stream from the contactor over a porous poly(tetrafluoroethylene) (PTFE) membrane. This occurs inside the separator chip. The porous PTFE membrane is hydrophobic and repels the aqueous slugs but allows the organic stream to pass through. A pressure differential across this membrane is controlled using back pressure regulators. As the mixture moves through the channel, the differential in pressure drives the organic phase through the membrane while the aqueous phase is retained (Figure S2). We hypothesized that we could use this module to remove high boiling water soluble solvent from the reaction mixture. Initially, we focused our efforts on combining a simple one chip reaction with microfluidic extraction unit (Figure S3). Product exiting from the reactor was mixed with excess water and dichloromethane (introduced by an external pump - AcuFlow Series III pump). Next this stream was introduced in to the liquid–liquid extractor unit. Organic and aqueous layer analyzed by LC–MS and showed that the extraction of the product to dichloromethane (DCM) layer is successful at 165 or 100 (see Figure S3 below) mbar cross membrane pressure. A back pressure of 3.0 bar was maintained on the aqueous outlet of the FLLEX, while the organic outlet's back pressure was maintained at a 165 or 100 mbar deficit (see Figure S3 below) to the aqueous outlet by way of the FLLEX module's internal pressure differential control valve.

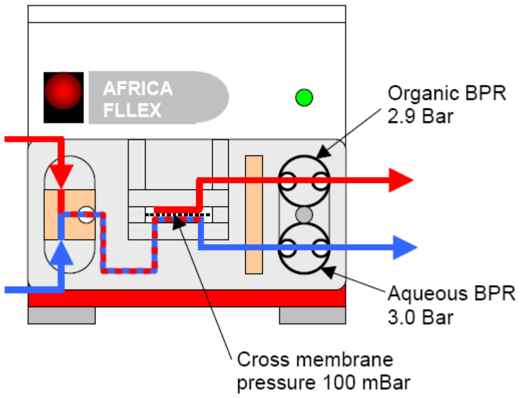

Figure S2: AFRICA® Fluidic Liquid-Liquid Extraction (FLLEX) Module.

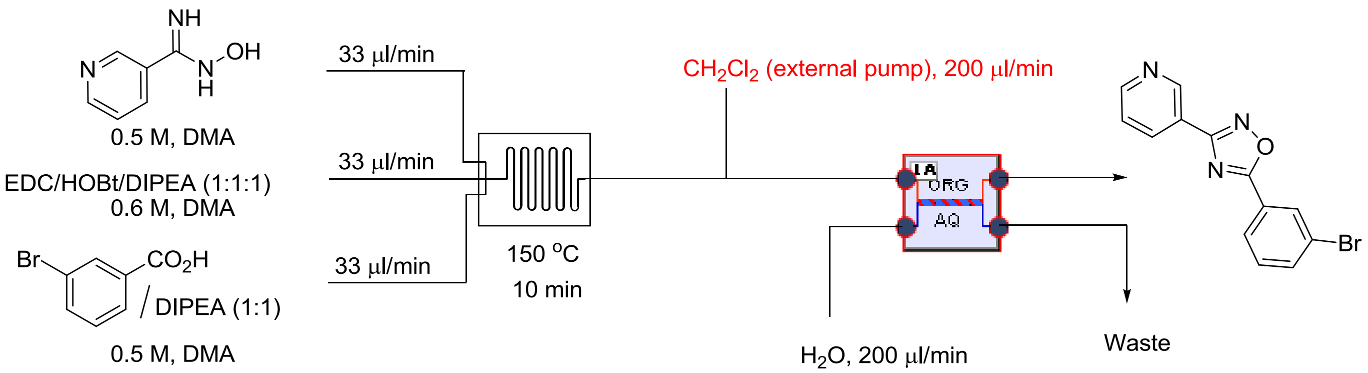

Figure S3: Flow synthesis of oxadiazoles in combination with microfluidic extraction.

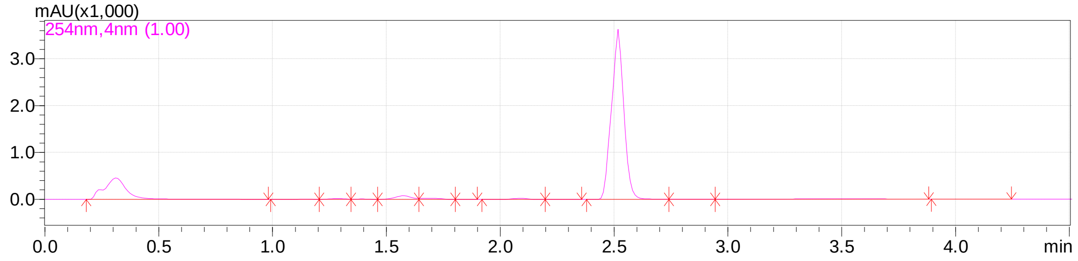

Crude

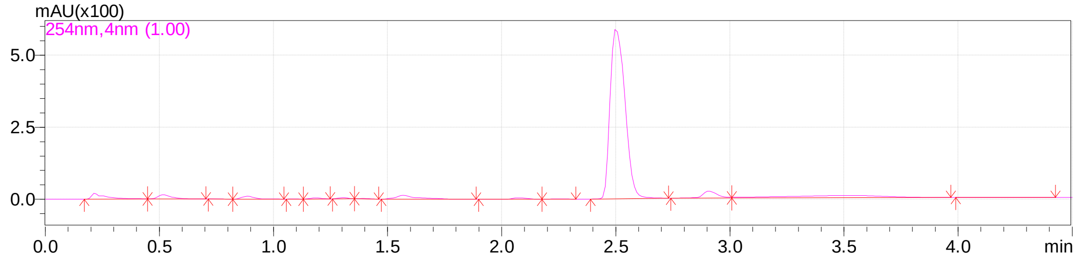

Organic layer

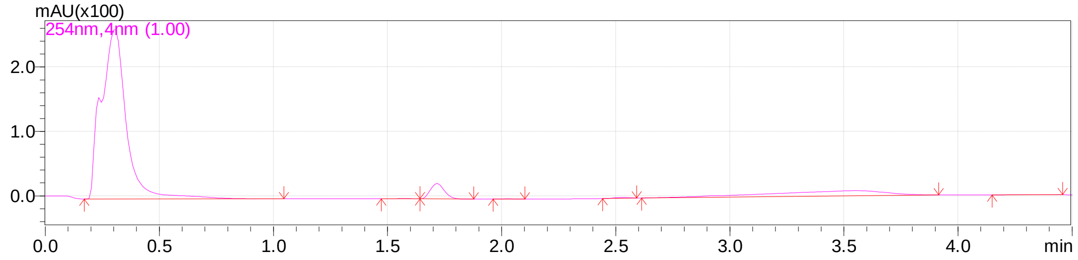

Aqueous layer

The Syrris AFRICA FLLEX module (FLLEX module controlled with a computer) was successfully combined with a Vapourtec system to remove solvent (DMA) and impurities from the reaction medium. Further experimentation showed that toluene is much better than CH_2Cl_2 for the extraction. On a preparative scale using the three reactor flow reaction setup (Schemes 2 and 3 of main article), the desired oxadiazole derivatives were obtained in good yield with a throughput of ≈0.5 g/h.

Procedures and data for the flow synthesis of 1,2,4-oxadiazoles in Table 2.

3-(4-Chlorophenyl)-5-(cyclopentylmethyl)-1,2,4-oxadiazole (Table 2, entry 1)

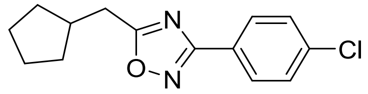

Following the general procedure the reaction mixture (2.5 mL) was collected, and this was purified using automated SiO_2 chromatography to provide the title compound as a yellow solid (70 mg, 81%). ^{1}H NMR (400 MHz, $CDCl_3$) δ 8.02 (m, 2H), 7.45 (m, 2H), 2.95 (dd, *J* = 2.3, 7.3 Hz, 2H), 2.41 (septet, *J* = 7.8 Hz, 1H), 1.89 (m, 2H), 1.64 (m, 4H), 1.30 (m, 2H); ^{13}C NMR (100 MHz, $CDCl_3$) δ 179.8, 167.4, 137.1, 129.1, 128.7, 125.5, 37.9, 32.4, 32.3, 24.9; HRMS (ESI) *m/z* calcd for $C_{14}H_{15}ClN_2O$ $[M+H]^+$ 263.0946, found 263.0947.

5-(3-Bromophenyl)-3-(4-nitrophenyl)-1,2,4-oxadiazole[1] (Table 2, entry 2)

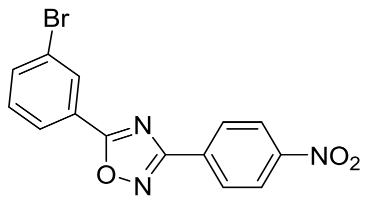

Following the general procedure, the reaction mixture (2.5 mL) was collected, and this was purified using automated SiO_2 chromatography to provide the title compound as a white solid (61 mg, 53%). ^{1}H NMR (400 MHz, $CDCl_3$) δ 8.36 (m, 5H), 8.14 (d, *J* = 7.8 Hz, 1H), 7.75 (d, *J* = 8.2 Hz, 1H), 7.44 (t, *J* = 7.8 Hz, 1H); ^{13}C NMR (100 MHz, $CDCl_3$) δ 175.1, 167.5, 149.6, 136.1, 132.5, 131.1, 130.8, 128.5, 126.7, 125.5, 124.1, 123.3; LCMS (ESI) *m/z* calcd for $C_{14}H_8BrN_3O_3$ $[M+H]^+$ 345.9749, found 346.00.

3-(3-(Pyridin-2-yl)-1,2,4-oxadiazol-5-yl)benzonitrile[2] (Table 2, entry 3)

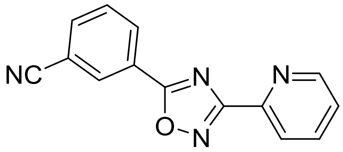

Following the general procedure, the reaction mixture (2.5 mL) was collected, and this was purified using automated SiO_2 chromatography to provide the title compound as a white solid (61 mg, 75%). ^{1}H NMR (400 MHz, $CDCl_3$) δ 8.81 (m, 1H), 8.54 (m, 1H), 8.46 (d, *J* = 7.8 Hz, 1H), 8.18 (d, *J* = 7.8 Hz, 1H), 7.86 (m, 2H), 7.68 (t, *J* = 8.7 Hz, 1H), 7.45 (m, 1H); ^{13}C NMR (100 MHz, $CDCl_3$) δ 174.7, 169.0, 150.5, 145.8, 138.0, 136.2, 132.4, 132.0, 130.5, 126.3, 125.5, 123.8, 117.6, 114.2; HRMS (ESI) *m/z* calcd for $C_{14}H_8N_4O$ $[M+H]^+$ 249.0698, found 249.0770.

3-(5-(3-Bromophenyl)-1,2,4-oxadiazol-3-yl)benzonitrile (Table 2, entry 4)

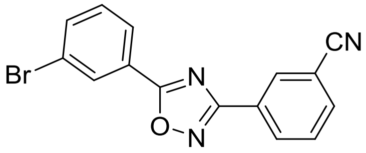

Following the general procedure, the reaction mixture (2.5 mL) was collected, and this was purified using automated SiO_2 chromatography to provide the title compound as a white solid (94 mg, 88%). ^{1}H NMR (400 MHz, $CDCl_3$) δ 8.49 (s, 1H), 8.40 (m, 2H), 8.16 (dd, *J* = 0.9, 7.8 Hz, 1H), 7.83 (m, 1H), 7.77 (m, 1H), 7.66 (t, *J* = 7.8 Hz, 1H), 7.46 (t, *J* = 7.8 Hz, 1H); ^{13}C NMR (100 MHz MHz, $CDCl_3$) δ 175.0, 167.4, 136.1, 134.5, 131.4, 131.11, 130.09, 130.8, 129.9, 128.1, 126.7, 125.6, 123.3, 118.0, 113.4; HRMS (ESI) *m/z* calcd for $C_{15}H_8BrN_3O$ $[M+H]^+$ 324.9924, found 324.9916.

5-(3-Bromophenyl)-3-(pyridin-2-yl)-1,2,4-oxadiazole[3] (Table 2, entry 5)

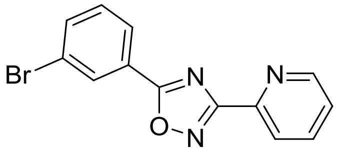

Following the general procedure, the reaction mixture (2.5 mL) was collected, and this was purified using automated SiO_2 chromatography to provide the title compound as a white solid (68 mg, 69%). ^{1}H NMR (400 MHz, $CDCl_3$) δ 8.83 (d, *J* = 5.0 Hz, 1H), 8.45 (s, 1H), 8.19 (m, 2H), 7.86 (dt, *J* = 1.8, 8.0 Hz, 1H), 7.72 (d, *J* = 7.8 Hz, 1H), 7.44 (m, 2H); ^{13}C NMR (100 MHz MHz, $CDCl_3$) δ 175.1, 168.9, 150.5, 146.2, 137.1, 135.9, 131.2, 130.6, 126.8, 125.7, 125.6, 123.3, 123.2; HRMS (ESI) *m/z* calcd for $C_{13}H_8BrN_3O$ $[M+H]^+$ 301.9852, found 301.9924.

5-(3-Bromophenyl)-3-(pyridin-3-yl)-1,2,4-oxadiazole[4] (Table 2, entry 6)

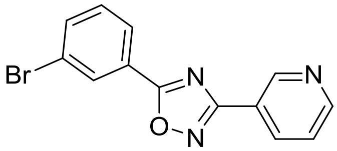

Following the general procedure, the reaction mixture (2.5 mL) was collected, and this was purified using automated SiO_2 chromatography to provide the title compound as a white solid (79 mg, 80%). ^{1}H NMR (400 MHz, $CDCl_3$) δ 9.37 (m, 1H), 8.75 (d, *J* = 1.4, 4.6 Hz, 1H), 8.41 (dt, *J* = 1.8, 8.2 Hz 1H), 8.36 (m, 1H), 8.13 (d, *J* = 7.8 Hz, 1H), 7.73 (m, 1H), 7.43 (m, 2H); ^{13}C NMR (100 MHz MHz, $CDCl_3$) δ 174.8, 167.1, 152.2, 148.7, 136.0, 134.7, 131.1, 130.7, 126.7, 125.7, 123.7, 123.2, 123.0; HRMS (ESI) *m/z* calcd for $C_{13}H_8BrN_3O$ $[M+H]^+$ 301.9853, found 301.9925.

5-(3-Bromophenyl)-3-(3-methoxyphenyl)-1,2,4-oxadiazole[5] (Table 2, entry 7)

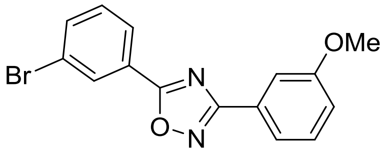

Following the general procedure, the reaction mixture (2.5 mL) was collected, and this was purified using automated SiO_2 chromatography to provide the title compound as a white solid (84 mg, 78%). ^{1}H NMR (400 MHz, $CDCl_3$) δ 8.36 (s, 1H), 8.13 (d, J = 7.8 Hz, 1H), 7.73 (m, 2H), 7.67 (s, 1H), 7.40 (m, 2H), 7.05 (dd, J = 2.8, 8.2 Hz, 1H), 3.89 (s, 3H); ^{13}C NMR (100 MHz MHz, $CDCl_3$) δ 174.3, 169.0, 159.9, 135.7, 131.0, 130.7, 130.0, 127.8, 126.6, 126.0, 123.1, 120.0, 117.9, 112.0, 55.5; HRMS (ESI) *m/z* calcd for $C_{15}H_{11}BrN_2O_2$ $[M+H]^+$ 331.0608, found 331.0607.

5-(Cyclopentylmethyl)-3-(isoquinolin-3-yl)-1,2,4-oxadiazole[6] (Table 2, entry 8)

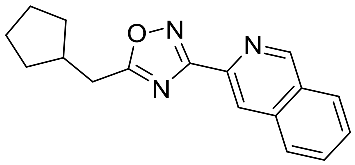

Following the general procedure, the reaction mixture (2.5 mL) was collected, and this was purified using automated SiO_2 chromatography to provide the title compound as a white solid (64 mg, 69%). ^{1}H NMR (400 MHz, $CDCl_3$) δ 9.37 (s, 1H), 8.51 (s, 1H), 8.02 (d, J = 7.8 Hz 1H), 7.92 (d, J = 8.2 Hz, 1H), 7.75 (t, J = 6.9 Hz, 1H), 7.68 (t, J = 6.9 Hz, 1H), 3.00 (m, 2H), 2.46 (septet, , J = 7.8 Hz, 1H), 1.89 (m, 2H), 1.62 (m, 4H), 1.32 (m, 2H); ^{13}C NMR (100 MHz MHz, $CDCl_3$) δ 180.2, 168.4, 153.3, 140.1, 135.8, 131.1, 129.3, 128.7, 127.8, 127.4, 120.6, 38.0, 32.5, 32.4, 24.9; HRMS (ESI) *m/z* calcd for $C_{17}H_{17}N_3O$ $[M+H]^+$ 280.1444, found 280.1430.

General procedure for the continuous flow synthesis of imidazo[1,2-*a*]pyridine-2-yl-1,2,4-oxadiazoles (Table 3).

5-(6-Bromoimidazo[1,2-*a*]pyridin-2-yl)-3-(4-chlorophenyl)-1,2,4-oxadiazole (Table 3, entry 1)

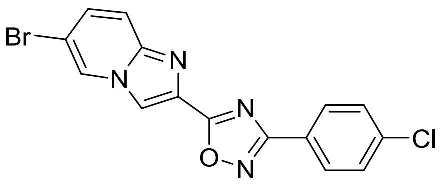

Following the general procedure, the reaction mixture (3.0 mL) was collected, purified using automated SiO_2 chromatography to provide the title compound as a yellow solid (51 mg, 46%). ^{1}H NMR (400 MHz, $CDCl_3$) δ 8.36 (s, 1H), 8.31 (s, 1H), 8.16 (s, 1H), 8.14 (s, 1H), 7.65 (d, *J* = 9.6 Hz, 1H), 7.48 (s, 1H), 7.46 (s, 1H), 7.37 (dd, *J* = 1.8, 9.6 Hz, 1H); ^{13}C NMR (100 MHz, $CDCl_3$) δ 171.2, 168.2, 144.6, 137.6, 131.7, 130.5, 129.2, 129.0, 126.2, 125.2, 119.4, 114.7, 109.4; HRMS (ESI) *m/z* calcd for $C_{15}H_8BrClN_4O$ $[M+H]^+$ 376.9621, found 376.9622.

3-(4-Chlorophenyl)-5-(6-methylimidazo[1,2-*a*]pyridin-2-yl)-1,2,4-oxadiazole (Table 3, entry 2)

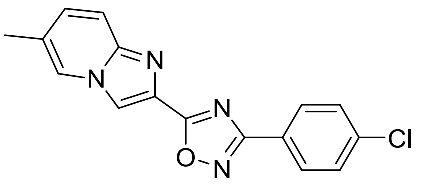

Following the general procedure, the reaction mixture (3.0 mL) was collected, purified using automated SiO_2 chromatography to provide the title compound as a yellow solid (30 mg, 32%). ^{1}H NMR (400 MHz, $CDCl_3$) δ 8.25 (s, 1H), 8.16 (m, 2H), 7.97 (s, 1H), 7.63 (d, *J* = 9.6 Hz, 1H), 7.40 (m, 2H), 7.15 (d, *J* = 9.2 Hz, 1H), 2.35 (s, 3H); ^{13}C NMR (100 MHz, $CDCl_3$) δ 172.1, 168.2, 145.3, 137.5, 130.6, 130.0, 129.1,

129.0, 125.3, 124.1, 123.6, 118.1, 114.5, 18.0; HRMS (ESI) *m/z* calcd for $C_{16}H_{11}ClN_4O$ $[M+H]^+$ 311.0694, found 311.0678.

5-(7-Chloroimidazo[1,2-*a*]pyridin-2-yl)-3-(4-chlorophenyl)-1,2,4-oxadiazole(Table 3, entry 3)

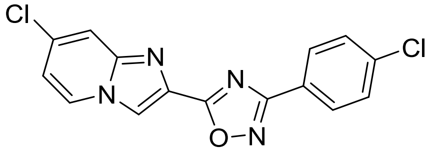

Following the general procedure, the reaction mixture (3.0 mL) was collected, purified using automated SiO_2 chromatography to provide the title compound as a yellow solid (43 mg, 44%). ^1H NMR (400 MHz, $CDCl_3$) δ 8.33 (s, 1H), 8.16 (s, 1H), 8.14 (s, 1H), 8.12 (s, 1H), 7.74 (s, 1H), 7.48 (s, 1H), 7.46 (s, 1H), 6.93 (dd, *J* = 1.8, 6.9 Hz, 1H); ^{13}C NMR (100 MHz, $CDCl_3$) δ 170.5, 138.4, 137.4, 134.6, 132.4, 129.1, 128.9, 128.5, 127.6, 127.4, 125.5, 111.6, 110.4; HRMS (ESI) *m/z* calcd for $C_{15}H_8Br_2N_4O$ $[M+H]^+$ 420.9118, found 420.9120.

5-(6-Bromoimidazo[1,2-*a*]pyridin-2-yl)-3-(4-bromophenyl)-1,2,4-oxadiazole(Table 3, entry 4)

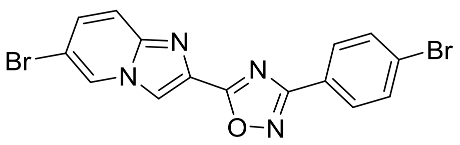

Following the general procedure, the reaction mixture (3.0 mL) was collected, purified using automated SiO_2 chromatography to provide the title compound as a yellow solid (52 mg, 42%). ^1H NMR (400 MHz, $CDCl_3$) δ 8.36 (s, 1H), 8.31 (s, 1H), 8.09 (s, 1H), 8.07 (s, 1H), 7.64 (m, 3H), 7.37 (d, *J* = 9.6 Hz, 1H); ^{13}C NMR (100 MHz, $CDCl_3$) δ 171.3, 168.4, 144.8, 132.1, 131.7, 130.5, 129.2, 126.1, 125.91, 125.89, 119.5, 114.9, 109.3; HRMS (ESI) *m/z* calcd for $C_{15}H_8Br_2N_4O$ $[M+H]^+$ 418.9066, found 418. 9127.

5-(6-Bromoimidazo[1,2-*a*]pyridin-2-yl)-3-(pyridin-2-yl)-1,2,4-oxadiazole (Table 3, entry 5)

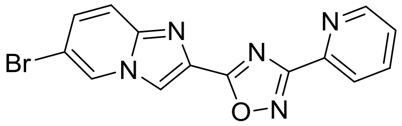

Following the general procedure, the reaction mixture (3.0 mL) was collected, purified using automated SiO_2 chromatography to provide the title compound as a yellow solid (56 mg, 55%). ^{1}H NMR (400 MHz, $CDCl_3$) δ 8.84 (d, *J* = 4.6 Hz, 1H), 8.43 (s, 1H), 8.40 (s, 1H), 8.30 (d, *J* = 7.8 Hz, 1H), 7.89 (dt, *J* = 1.8, 7.8 Hz, 1H), 7.67 (d, *J* = 9.6 Hz, 1H), 7.47 (m, 1H), 7.39 (dd, *J* = 1.8, 9.6 Hz, 1H); ^{13}C NMR (100 MHz, $CDCl_3$) δ 171.8, 168.7, 150.4, 146.3, 144.6, 137.1, 130.5, 126.2, 125.6, 123.5, 119.5, 115.2, 109.2; HRMS (ESI) *m/z* calcd for $C_{14}H_8BrN_5O$ $[M+H]^+$ 341.9985, found 341.9980.

5-(6-Bromoimidazo[1,2-*a*]pyridin-2-yl)-3-(pyridin-3-yl)-1,2,4-oxadiazole (Table 3, entry 6)

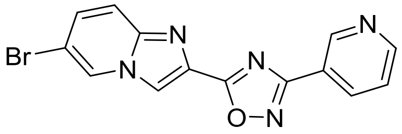

Following the general procedure, the reaction mixture (3.0 mL) was collected, purified using automated SiO_2 chromatography to provide the title compound as a yellow solid (60 mg, 59%). ^{1}H NMR (400 MHz, $CDCl_3$) δ 9.45 (s, 1H), 8.78 (m, 1H), 8.50 (d, *J* = 8.2 Hz, 1H), 8.40 (s, 1H), 8.36 (s, 1H), 7.68 (d, *J* = 9.6 Hz, 1H), 7.47 (m, 1H), 7.41 (d, *J* = 9.6 Hz, 1H); ^{13}C NMR (100 MHz, $CDCl_3$) δ 171.6, 167.1, 152.1, 148.8, 144.6, 135.0, 131.5, 130.6, 126.1, 123.7, 123.0,119.5, 115.0, 109.3; HRMS (ESI) *m/z* calcd for $C_{14}H_8BrN_5O$ $[M+H]^+$ 341.9985, found 341.9976.

3-(4-Chlorophenyl)-5-(imidazo[2,1-*b*]thiazol-6-yl)-1,2,4-oxadiazole (Table 3, entry 7)

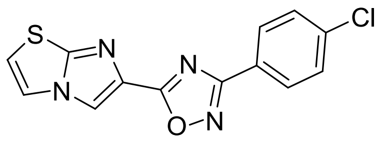

Following the general procedure, the reaction mixture (3.0 mL) was collected, purified using automated SiO_2 chromatography to provide the title compound as a yellow solid (60 mg, 59%). ^{1}H NMR (400 MHz, DMSO-d_6) δ 8.75 (d, *J* = 3.2 Hz, 1H), 8.08 (m, 2H), 8.03 (dd, *J* = 2.8, 4.6 Hz, 1H), 7.68 (m, 2H), 7.50 (dd, *J* = 2.8, 4.1 Hz, 1H); ^{13}C NMR (100 MHz, DMSO-d_6) δ 171.7, 167.1, 151.3, 136.4, 131.2, 129.4, 128.9, 125.1, 120.1, 117.6; HRMS (ESI) *m/z* calcd for $C_{13}H_7ClN_4OS$ $[M+H]^+$ 303.0102, found 303.0099.

5-(6-Bromoimidazo[1,2-*a*]pyridin-2-yl)-3-(3-methoxyphenyl)-1,2,4-oxadiazole (Table 3, entry 8)

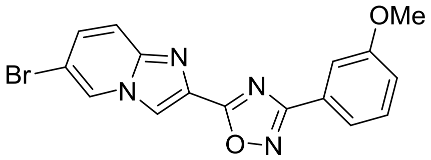

Following the general procedure, the reaction mixture (3.0 mL) was collected, purified using automated SiO_2 chromatography to provide the title compound as a yellow solid (39 mg, 35%). ^{1}H NMR (400 MHz, $CDCl_3$) δ 8.36 (s, 1H), 8.31 (s, 1H), 7.80 (d, *J* = 7.3 Hz, 1H), 7.73 (m, 1H), 7.65 (d, *J* = 9.6 Hz, 1H), 7.38 (m, 2H), 7.06 (dd, *J* = 2.3, 8.2 Hz, 1H), 3.88 (s, 3H); ^{13}C NMR (100 MHz, $CDCl_3$) δ 171.1, 169.0, 159.9, 144.6, 131.8, 130.4, 129.9, 127.8, 126.1, 120.1, 119.4, 118.1, 114.8, 112.0, 109.2, 55.5; HRMS (ESI) *m/z* calcd for $C_{16}H_{11}BrN_4O_2$ $[M+H]^+$ 371.0138, found 371.0119.

3-(5-(6-Bromoimidazo[1,2-*a*]pyridin-2-yl)-1,2,4-oxadiazol-3-yl)benzonitrile (Table 3, entry 9)

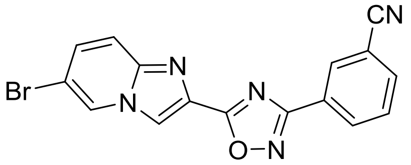

Following the general procedure, the reaction mixture (3.0 mL) was collected, purified using automated SiO_2 chromatography to provide the title compound as a white solid (38 mg, 35%). ^1H NMR (400 MHz, DMSO-d_6) δ 9.03 (s, 1H), 8.85 (s, 1H), 8.46 (s, 1H), 8.41 (d, *J* = 8.7 Hz 1H), 8.12 (d, *J* = 7.8 Hz, 1H), 7.84 (d, *J* = 7.8 Hz, 1H), 7.75 (d, *J* = 9.6 Hz, 1H), 7.58 (d, *J* = 9.6 Hz, 1H); HRMS (ESI) *m/z* calcd for $C_{16}H_8BrN_5O$ $[M+H]^+$ 365.9985, found 365.9983.

5-(6-Bromoimidazo[1,2-*a*]pyridin-2-yl)-3-(3,5-difluorophenyl)-1,2,4-oxadiazole (Table 3, entry 10)

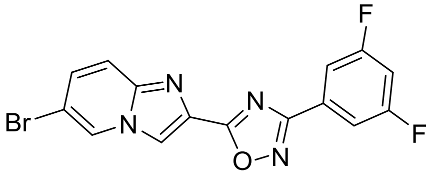

Following the general procedure, the reaction mixture (3.0 mL) was collected, purified using automated SiO_2 chromatography to provide the title compound as a white solid (33 mg, 29%). ^1H NMR (400 MHz, $CDCl_3$) δ 8.37 (s, 1H), 8.32 (s, 1H), 7.76 (d, *J* = 6.9 Hz, 2H), 7.65 (d, *J* = 10.0 Hz, 1H), 7.39 (d, *J* = 9.6 Hz, 1H), 6.96 (t, *J* = 8.2 Hz, 1H); ^{13}C NMR (100 MHz, $CDCl_3$) δ 171.7, 167.5, 164.5, 161.8, 144.7, 130.6, 126.2, 119.3, 115.1, 110.9, 110.7, 109.4, 106.7; HRMS (ESI) *m/z* calcd for $C_{15}H_7BrF_2N_4O$ $[M+H]^+$ 376.9844, found 376.9846.

5-(8-Chloro-6-(trifluoromethyl)imidazo[1,2-*a*]pyridin-2-yl)-3-(2-chlorophenyl)-1,2,4-oxadiazole[7] (Table 3, entry 11)

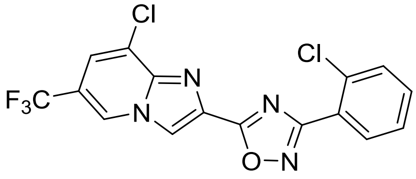

Following the general procedure, the reaction mixture (3.0 mL) was collected, purified using automated SiO_2 chromatography to provide the title compound as a white solid (24 mg, 20%). ^{1}H NMR (400 MHz, $CDCl_3$) δ 8.54 (s, 1H), 8.53 (s, 1H), 8.05 (d, J = 7.3 Hz, 1H), 7.55 (m, 2H), 7.42 (m, 2H); ^{13}C NMR (100 MHz, $CDCl_3$) δ 170.0, 167.9, 143.5, 133.6, 133.2, 132.0, 131.9, 130.9, 126.9, 126.1, 125.7, 123.8, 121.4, 121.0, 119.0,118.6, 117.7; HRMS (ESI) *m/z* calcd for $C_{16}H_7Cl_2F_3N_4O$ $[M+H]^+$ 398.9950, found 399.0024.

3-(2-Bromo-4-fluorophenyl)-5-(8-chloro-6-(trifluoromethyl)imidazo[1,2-*a*]pyridin-2-yl)-1,2,4-oxadiazole[7] (Table 3, entry 12)

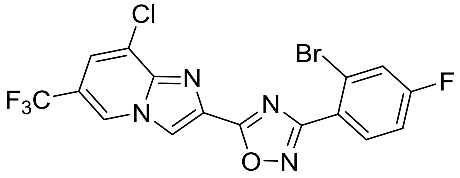

Following the general procedure, the reaction mixture (3.0 mL) was collected, purified using automated SiO_2 chromatography to provide the title compound as a white solid (34 mg, 25%). ^{1}H NMR (400 MHz, $CDCl_3$) δ 8.57 (s, 1H), 8.55 (s, 1H), 8.01 (dd, J = 5.9, 8.7 Hz, 1H), 7.57 (s, 1H), 7.52 (dd, J = 2.8, 7.8 Hz, 1H), 7.19 (dt, J = 2.8, 7.8 Hz, 1H); ^{13}C NMR (100 MHz, $CDCl_3$) δ 170.1, 168.0, 164.7, 162.2, 143.6, 133.7, 133.6, 133.0, 126.1, 124.2, 123.9, 123.0, 122.9, 121.8, 121.6, 121.0, 119.0, 118.7, 117.8, 115.0, 114.8; HRMS (ESI) *m/z* calcd for $C_{16}H_6BrClF_4N_4O$ $[M+H]^+$ 460.9351, found 460.9424.

References

1. Compound is commercially available.
2. Grant, D.; Dahl, R.; Cosford, N. D. P. *J. Org. Chem*. **2008**, *73(18)*, 7219-7223.
3. Raboisson, P.; Breitholtz-Emanuelsson, A.; Dahlloef, H.; Edwards, L.; Heaton, W. L.; Isaac, M.; Jarvie, K.; Kers, A.; Minidis, A.r B. E.; Nordmark, A.; et al. *Bioorg. Med. Chem. Lett.* **2012**, *22(22),* 6974-6979.
4. Gopalakrishnan, M.; Honore, M. P.; Lee, C.-H.; Malysz, J.n; Ji, J.; Li, T.; Schrimpf, M.l R.; Sippy, K. B.; Anderson, D. J. Oxadiazole derivatives as neuronal nicotinic acetylcholine receptor ligands and α4β2 pos. allosteric modulators and their preparation, pharmaceutical compositions and use in the treatment of diseases. WO 2008073942 A2 Jun 19, 2008 WO 2007-US87090 Dec 12, 2007.
5. Compound is commercially available.
6. Compound is commercially available.
7. Bannen, L.; Chan, D. S.-M.; Gu, X.-H.; Mac, M. B.; Ng, S.; Wang, T.-L.; Wang, Y.; Xu, W. Imidazo[1,2-*a*]pyridine derivatives, their use as S1P1 agonists and methods for their production. WO 2010065760 A1 Jun 10, 2010 WO 2009-US66620 Dec 3, 2009.

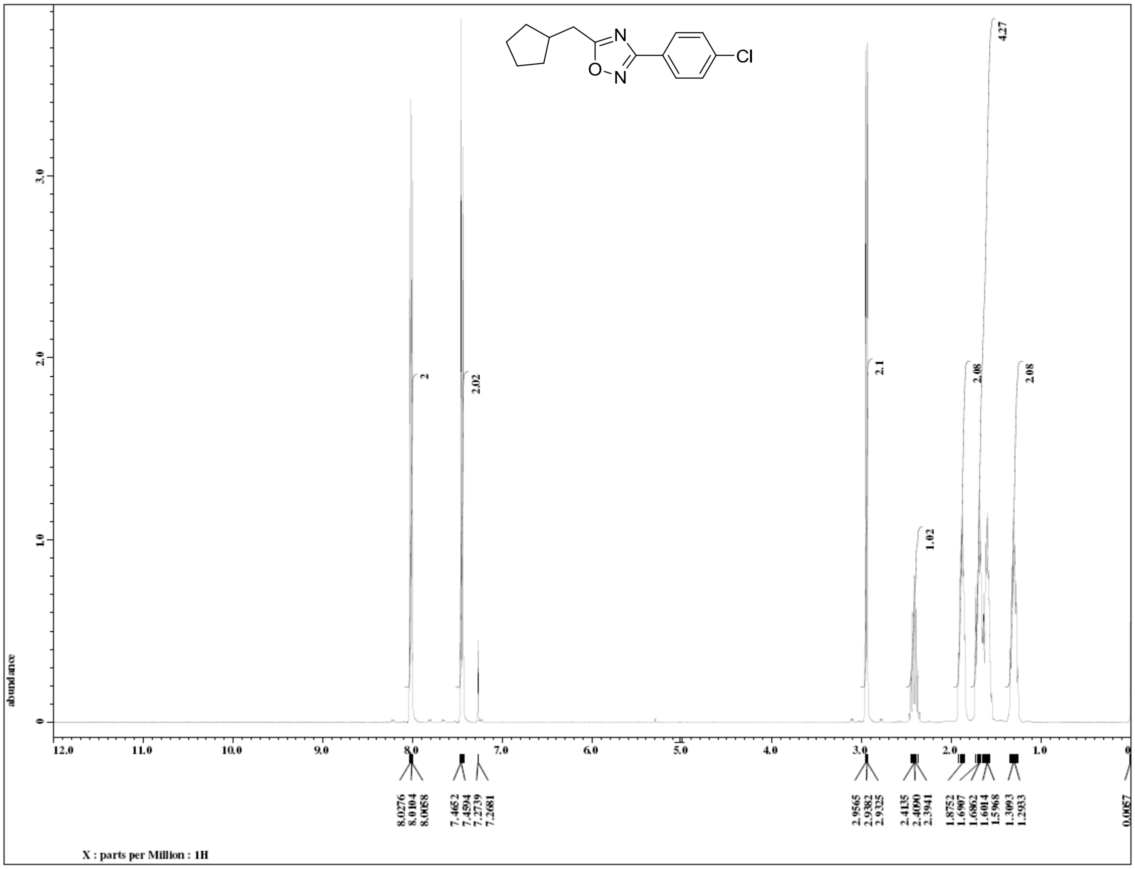
Cl
O
N
N
abundance
X : parts per Million : 1H
2
2.02
2.1
1.02
2.08
4.27
2.08
8.0276
8.0104
8.0058
7.4652
7.4594
7.2739
7.2681
2.9565
2.9382
2.9325
2.4135
2.4000
2.3941
1.8752
1.6907
1.6862
1.6014
1.5968
1.3093
1.2933
0.0057

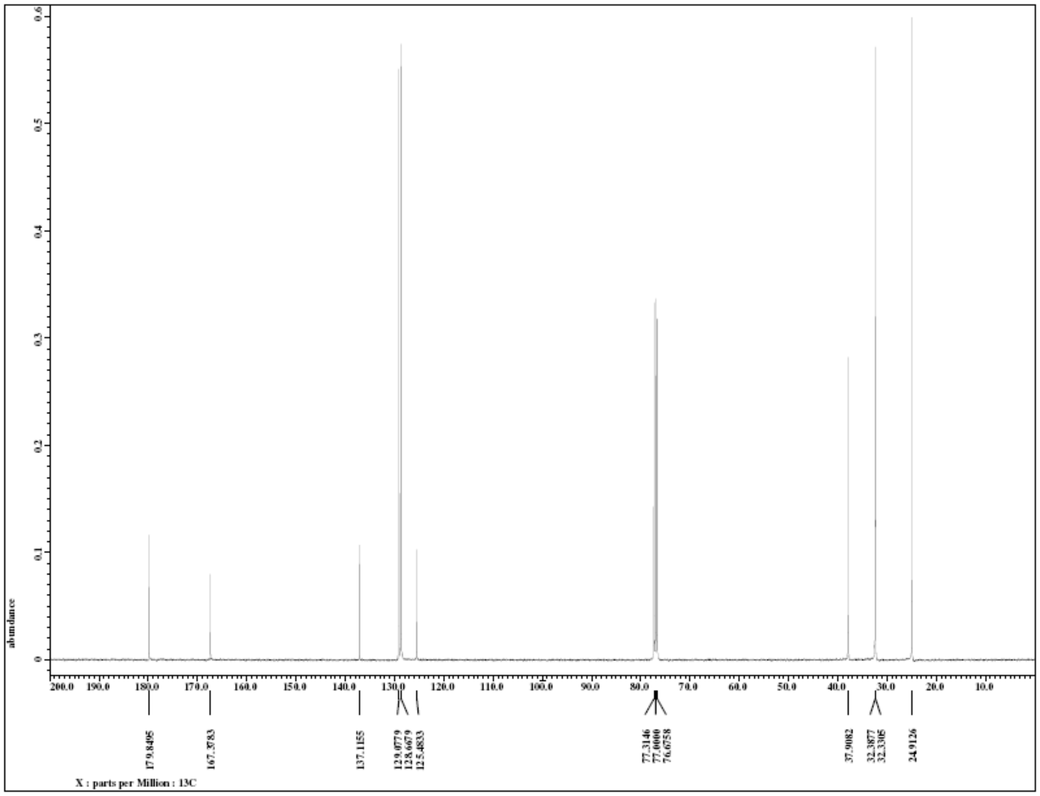
abundance
X : parts per Million : 13C
179.8495
167.3783
137.1155
129.0779
128.6679
125.4833
77.3146
77.0000
76.6758
37.9082
32.3877
32.3305
24.9126

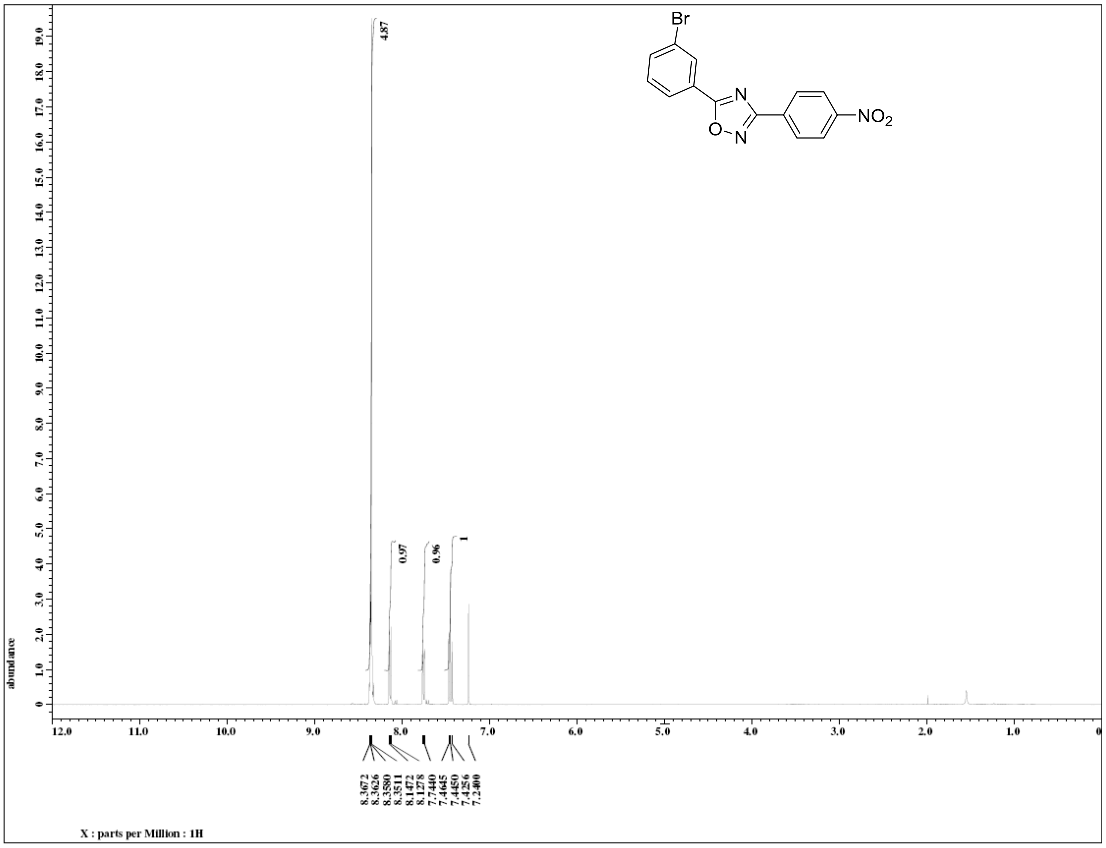
Br
N
O
N
NO2
abundance
4.87
0.97
0.96
1
8.3672
8.3626
8.3580
8.3511
8.1472
8.1278
7.7440
7.4645
7.4450
7.4256
7.2400
X : parts per Million : 1H

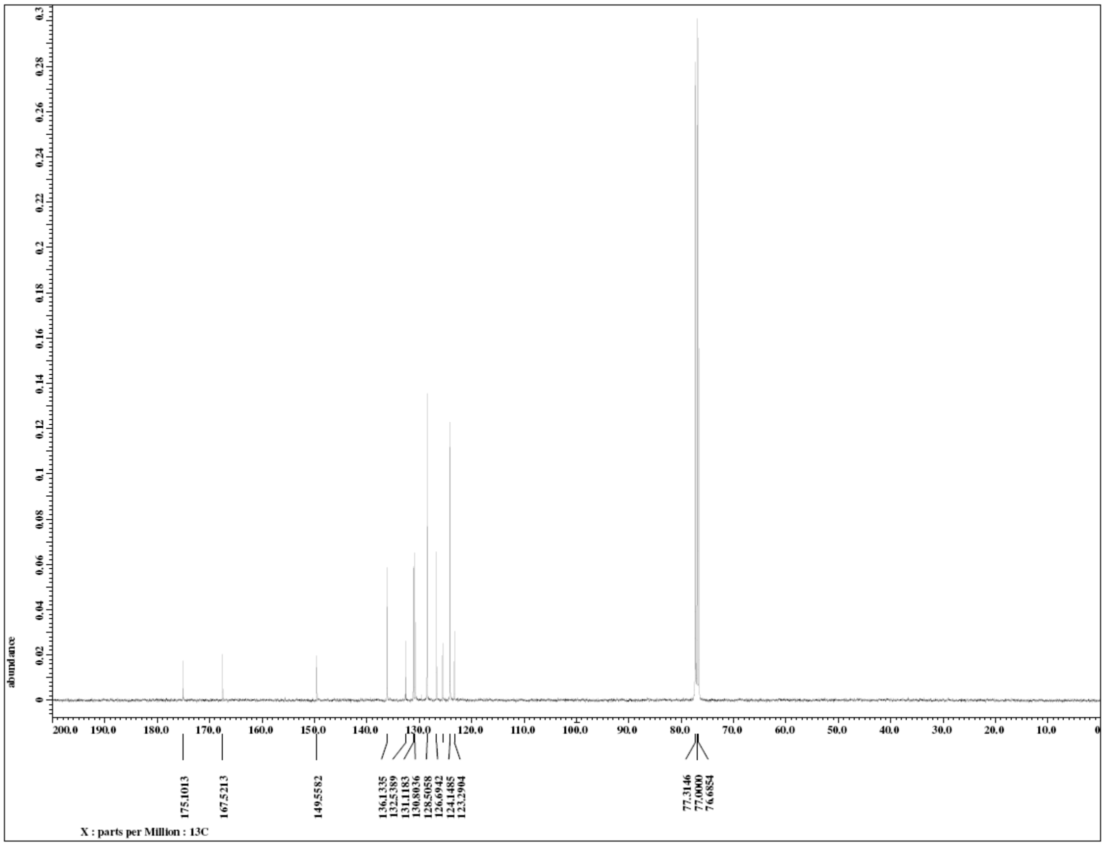
abundance
175.1013
167.5213
149.5582
136.1335
132.5389
131.1183
130.8036
128.5058
126.6942
124.1485
123.2904
77.3146
77.0000
76.6854
X : parts per Million : 13C

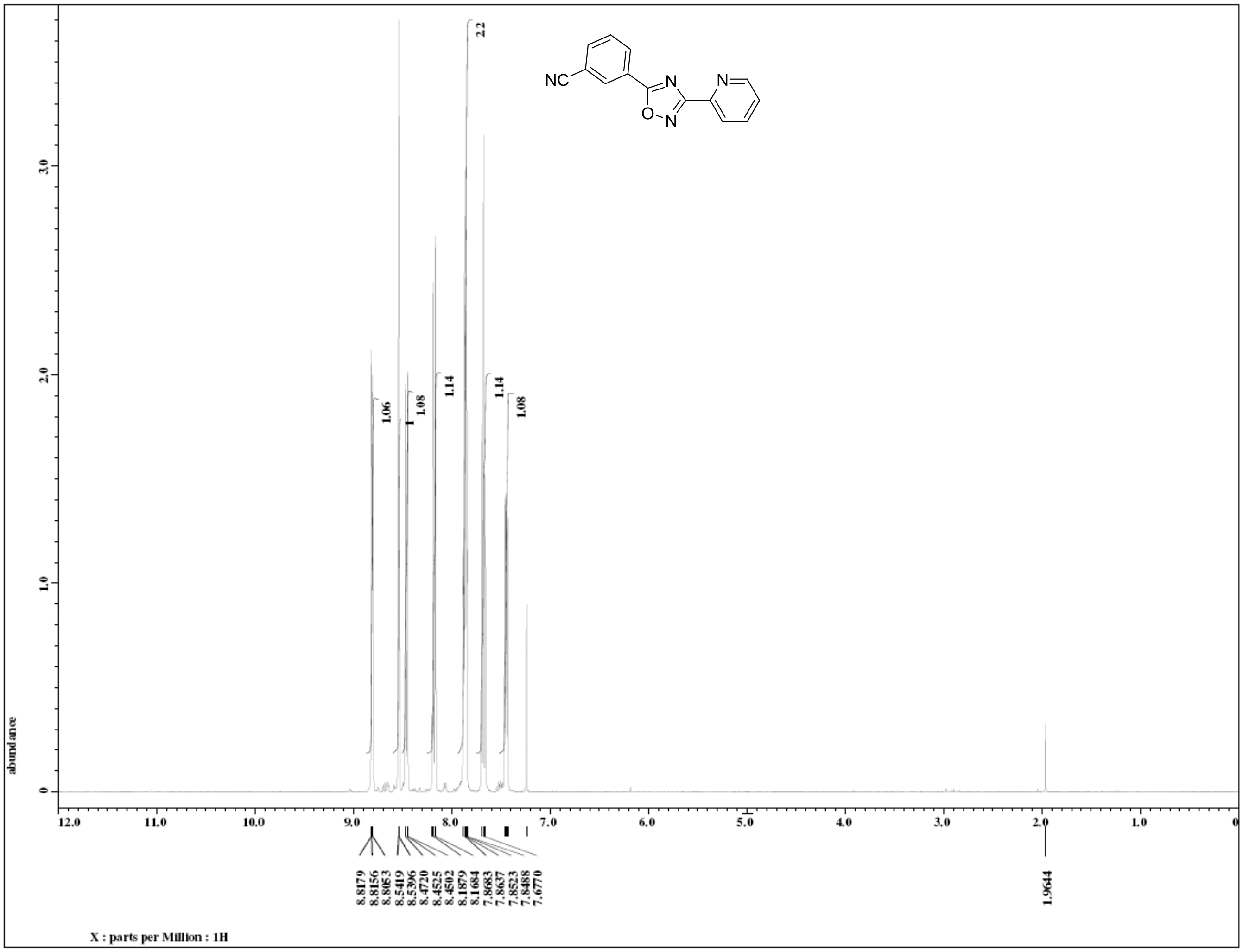
NC
O
N
N
N
abundance
X : parts per Million : 1H
1.06
1
1.08
1.14
2.2
1.14
1.08
8.8179
8.8156
8.8053
8.5419
8.5396
8.4720
8.4525
8.4502
8.1879
8.1684
7.8683
7.8637
7.8523
7.8488
7.6770
1.9644

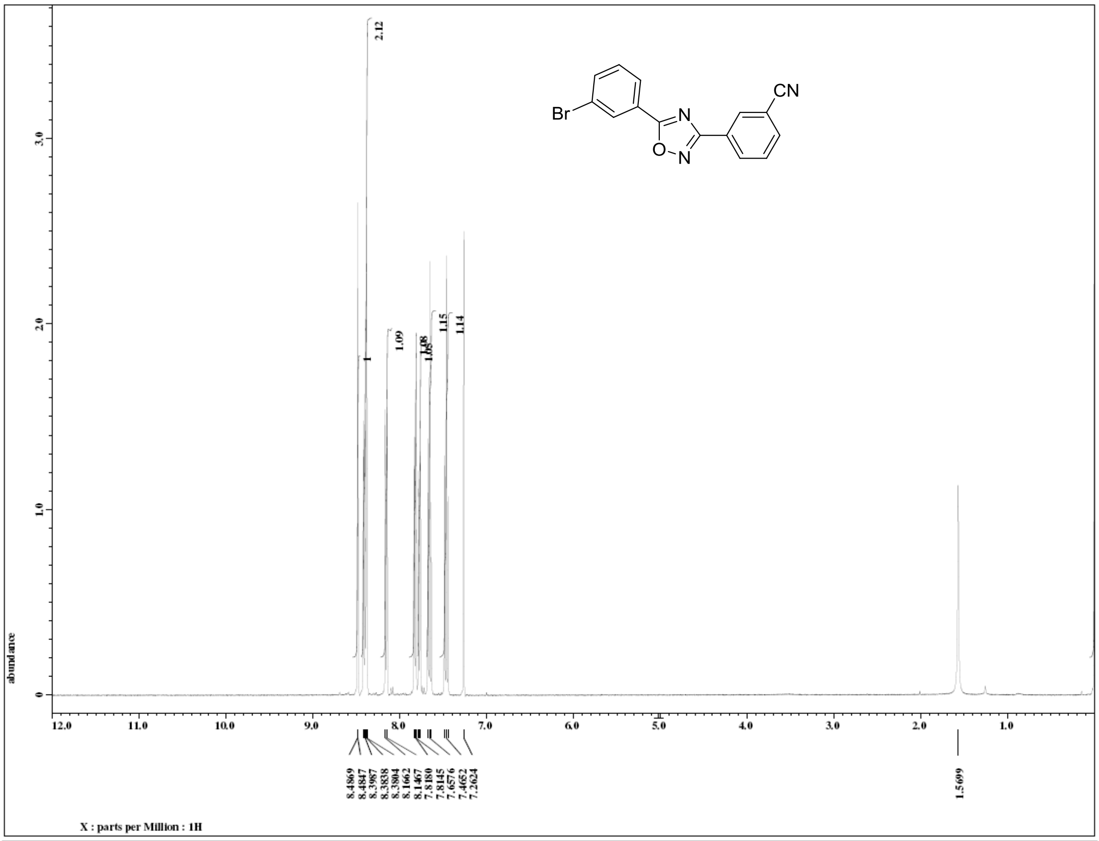

Br
CN
N
O
N
abundance
X : parts per Million : 1H
2.12
1.09
1.08
1.05
1.15
1.14
8.4869
8.4847
8.3987
8.3838
8.3804
8.1662
8.1467
7.8180
7.8145
7.6576
7.4652
7.2624
1.5609

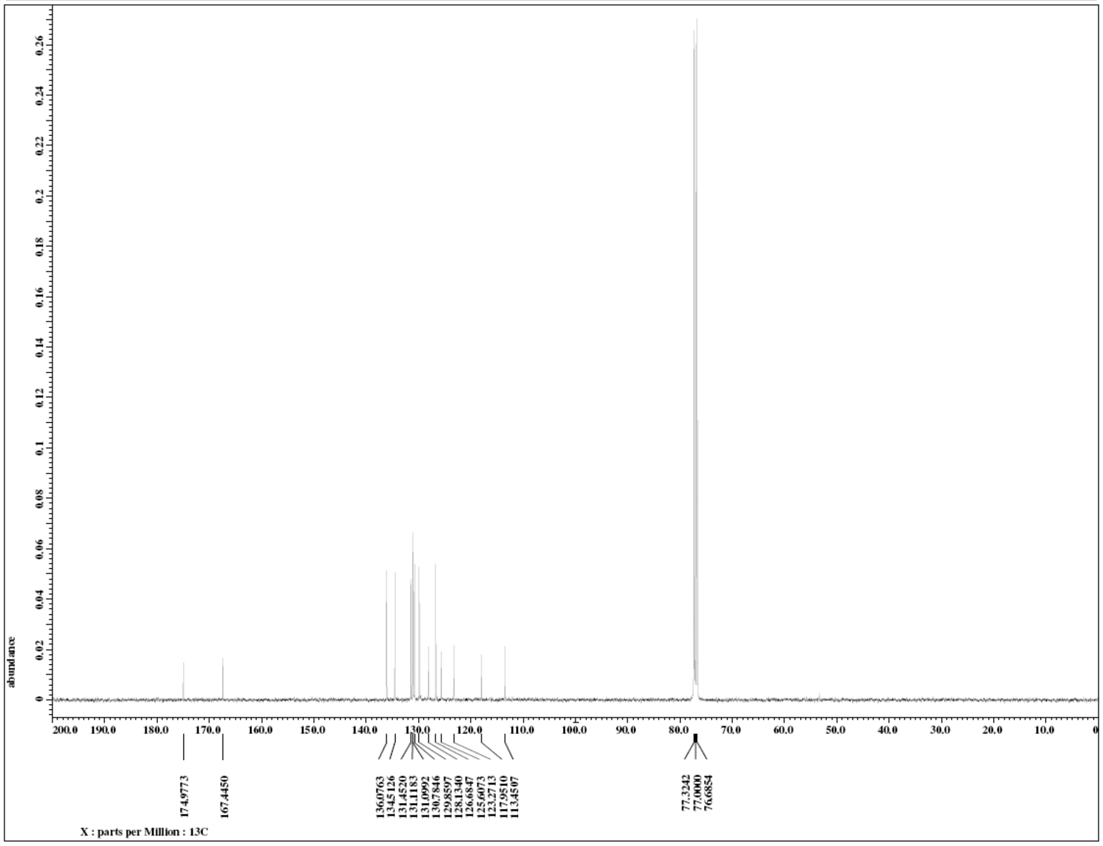

abundance
X : parts per Million : 13C
174.9773
167.4450
136.0763
134.5126
131.4520
131.1183
131.0992
130.7846
129.8597
128.1340
126.6847
125.6073
123.2713
117.9510
113.4507
77.3242
77.0000
76.6854

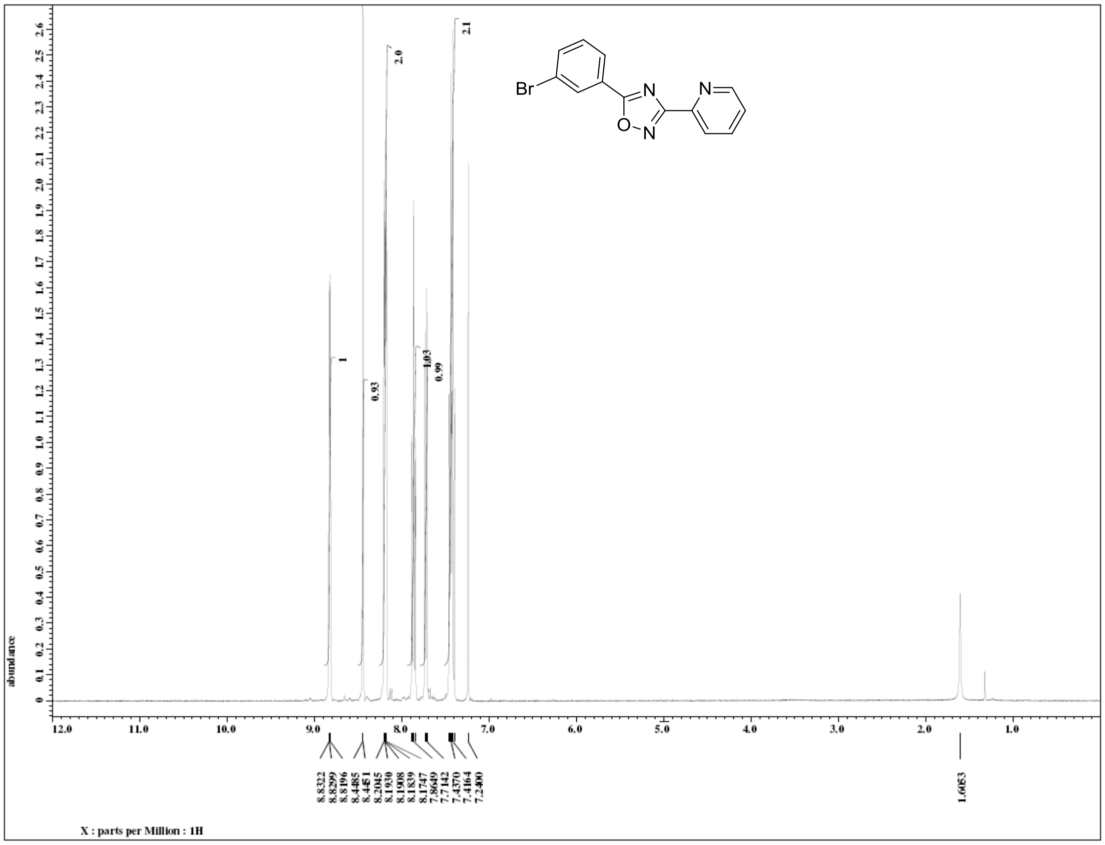
Br
N
O
N
N
abundance
1
0.93
2.0
1.03
0.99
2.1
8.8322
8.8299
8.8196
8.4485
8.4451
8.2045
8.1930
8.1908
8.1839
8.1747
7.8649
7.7142
7.4370
7.4164
7.2400
1.6053
X : parts per Million : 1H

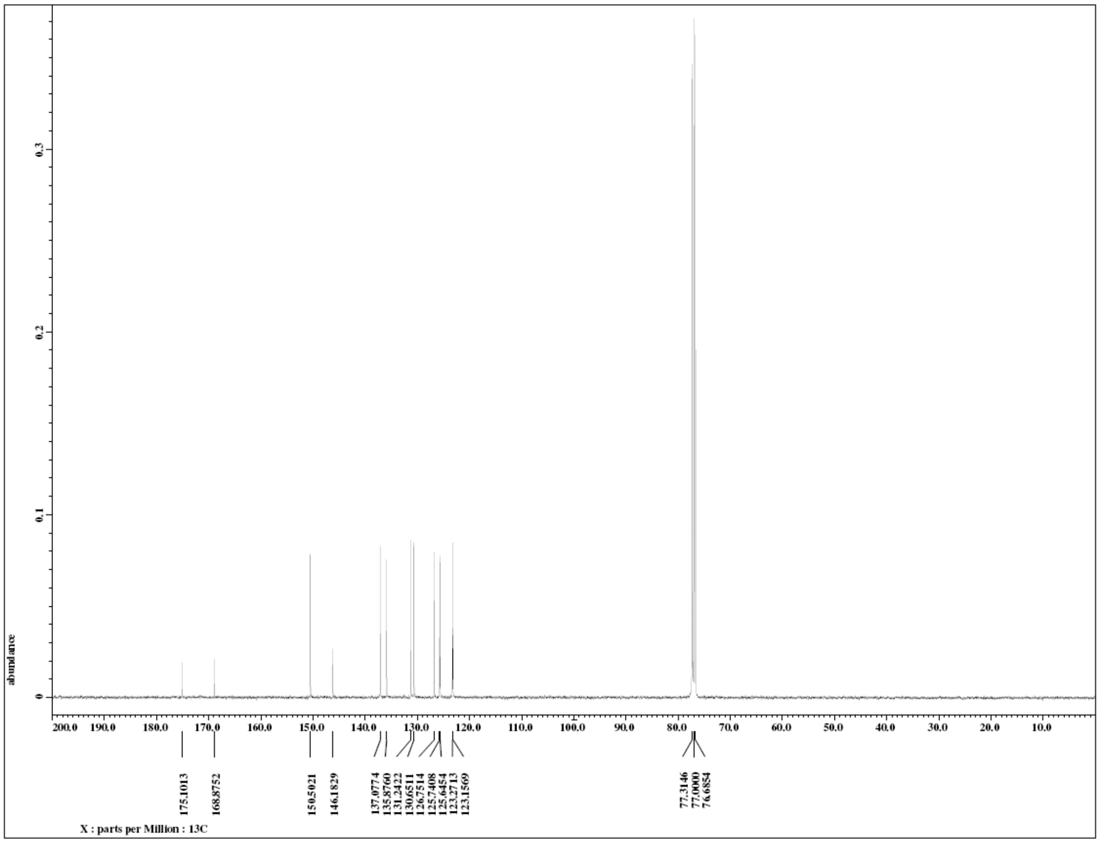
abundance
175.1013
168.8752
150.5021
146.1829
137.0774
135.8760
131.2422
130.6511
126.7514
125.7408
125.6454
123.2713
123.1569
77.3146
77.0000
76.6854
X : parts per Million : 13C

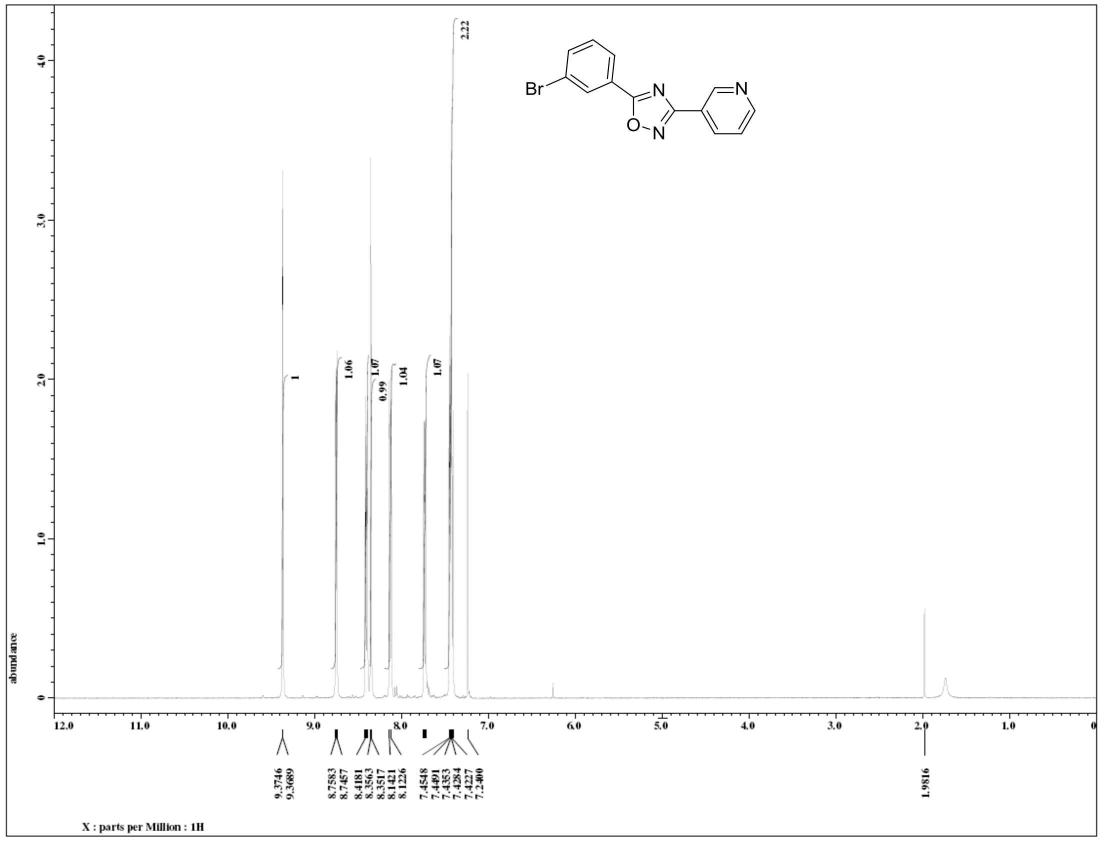
Br
N
O
N
N
2.22
1
1.06
1.07
0.99
1.04
1.07
abundance
9.3746
9.3689
8.7583
8.7457
8.4181
8.3563
8.3517
8.1421
8.1226
7.4548
7.4491
7.4353
7.4284
7.4227
7.2400
1.9816
X : parts per Million : 1H

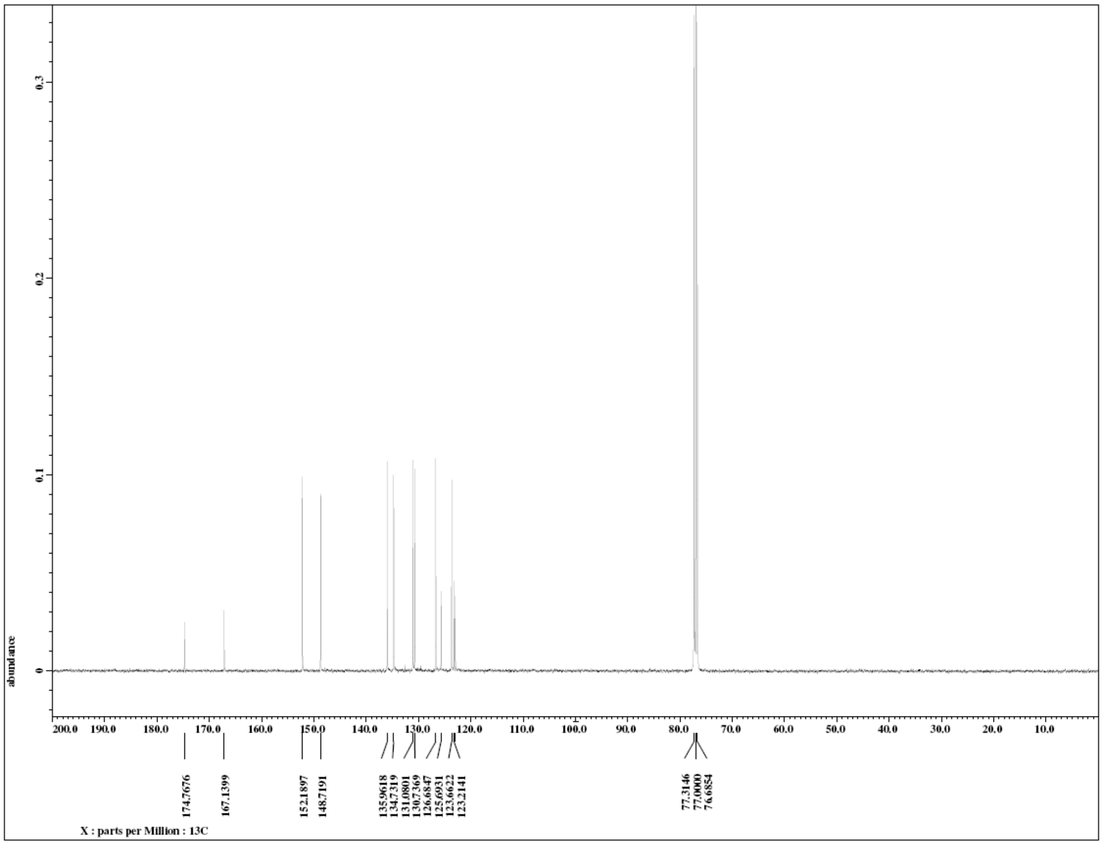
abundance
174.7676
167.1399
152.1897
148.7191
135.9618
134.7319
131.0801
130.7369
126.6847
125.6931
123.6622
123.2141
77.3146
77.0000
76.6854
X : parts per Million : 13C

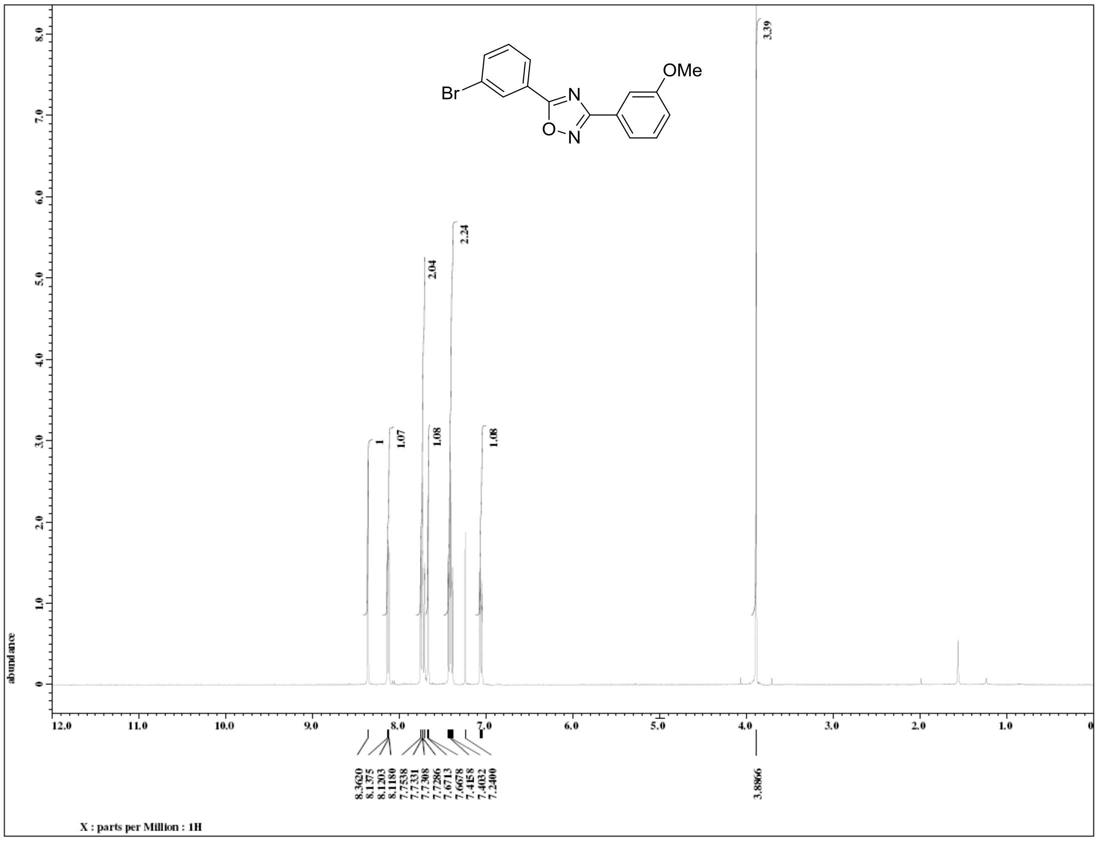
OMe
Br
N
O
N
3.39
2.24
2.04
1
1.07
1.08
1.08
abundance
8.3620
8.1375
8.1203
8.1180
7.7538
7.7331
7.7308
7.7286
7.6713
7.6678
7.4158
7.4032
7.2400
3.8866
X : parts per Million : 1H

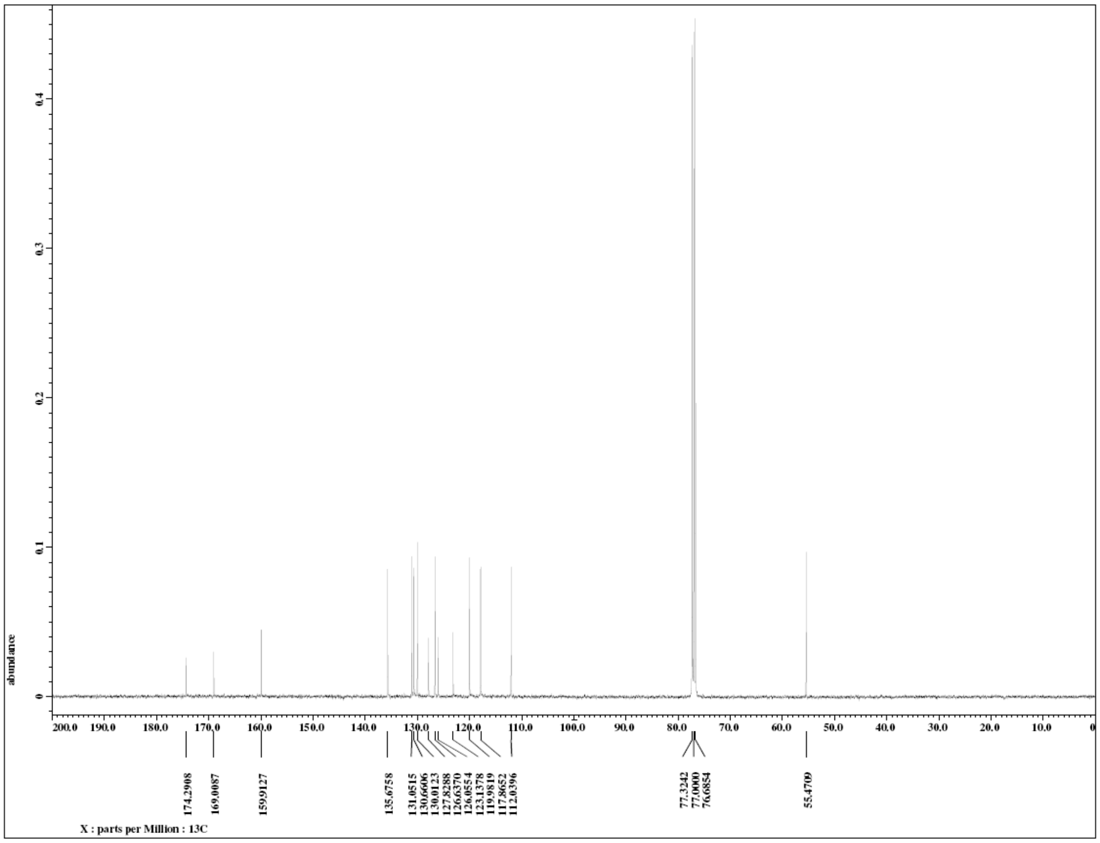
abundance
174.2908
169.0087
159.9127
135.6758
131.0515
130.6606
130.0123
127.8288
126.6370
126.0554
123.1378
119.9819
117.8652
112.0396
77.3242
77.0000
76.6854
55.4709
X : parts per Million : 13C

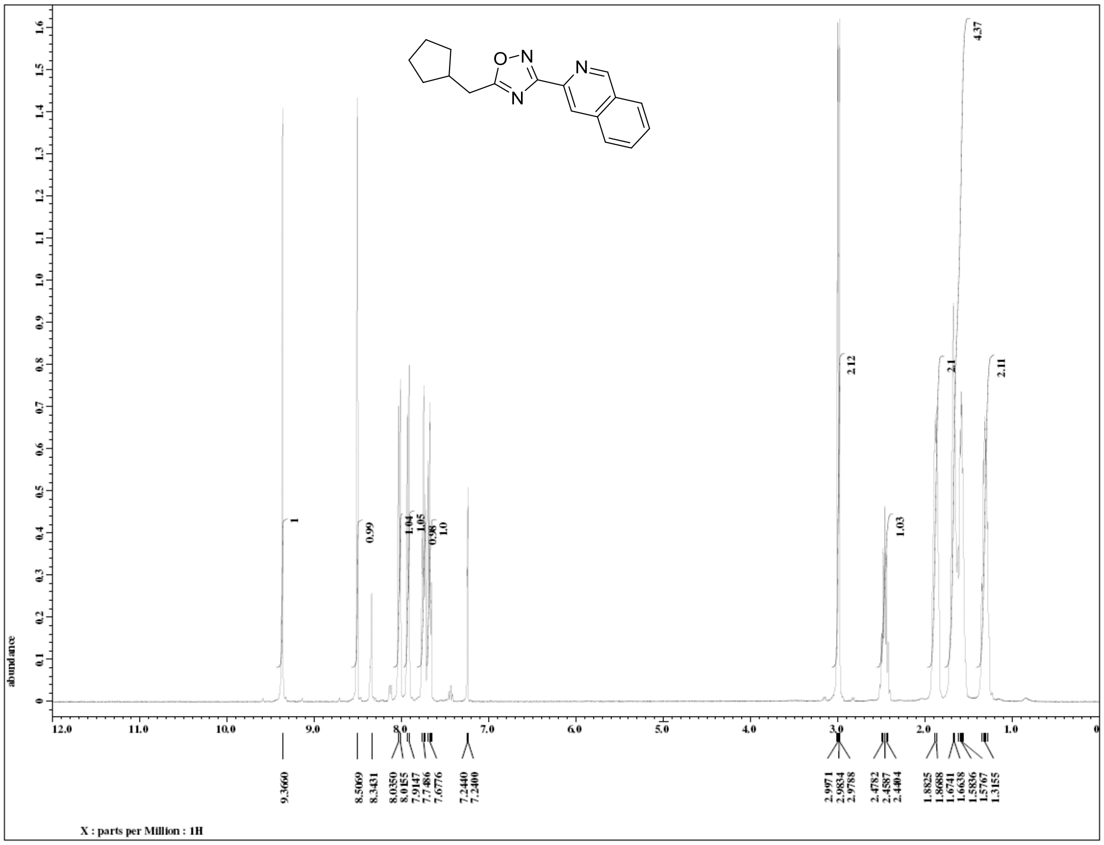

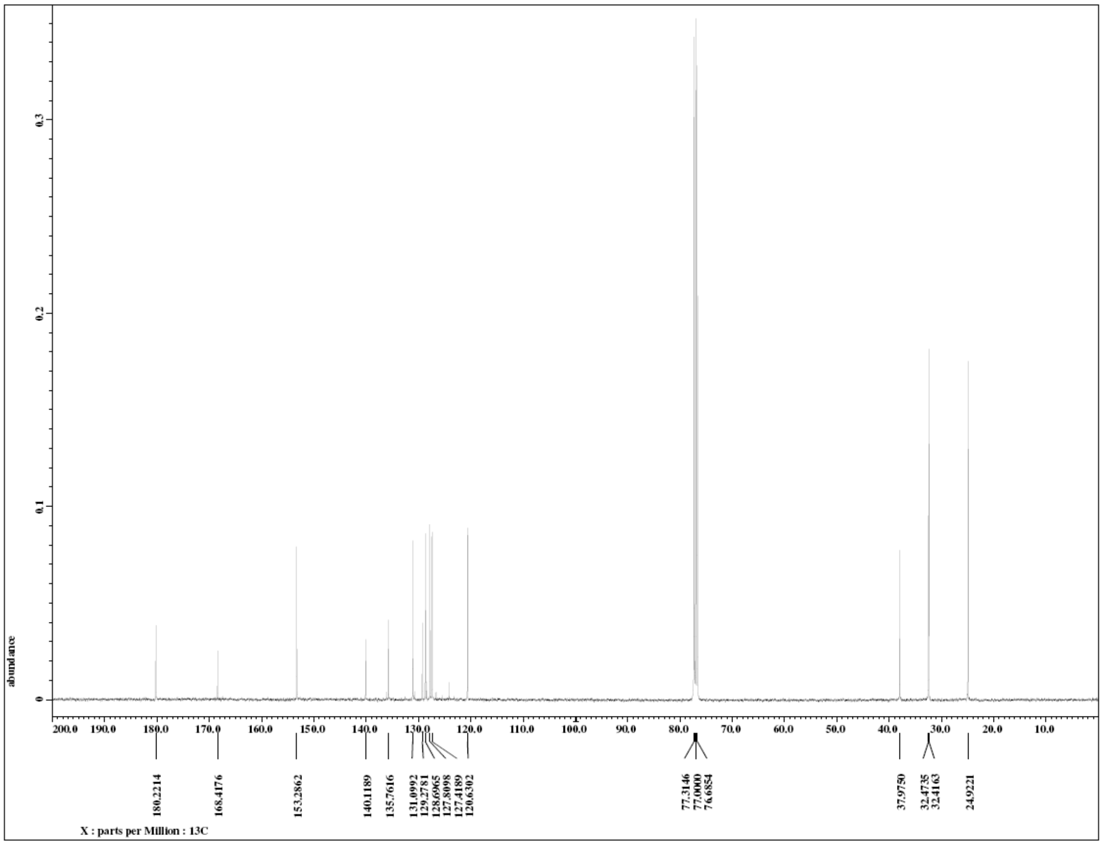

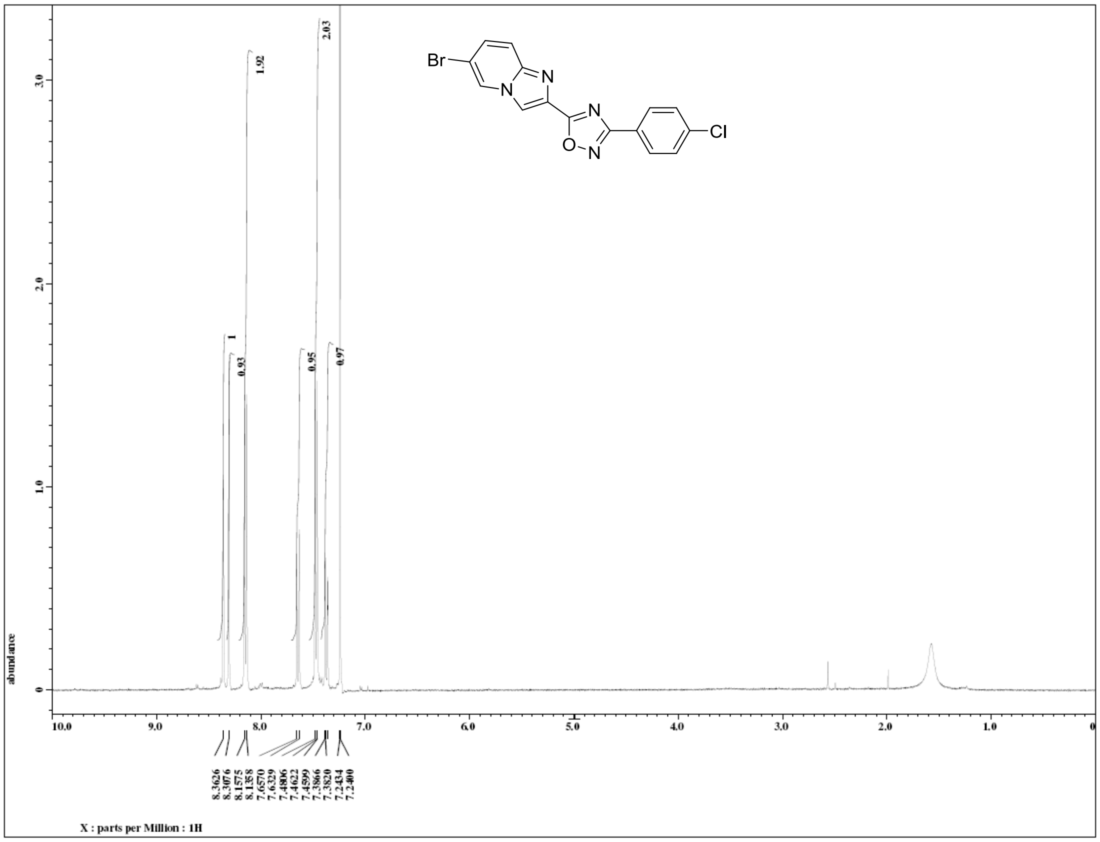
Br
N
N
N
O
N
Cl
abundance
1.92
2.03
1
0.93
0.95
0.97
8.3626
8.3076
8.1575
8.1358
7.6570
7.6329
7.4806
7.4622
7.4599
7.3866
7.3820
7.2434
7.2400
X : parts per Million : 1H

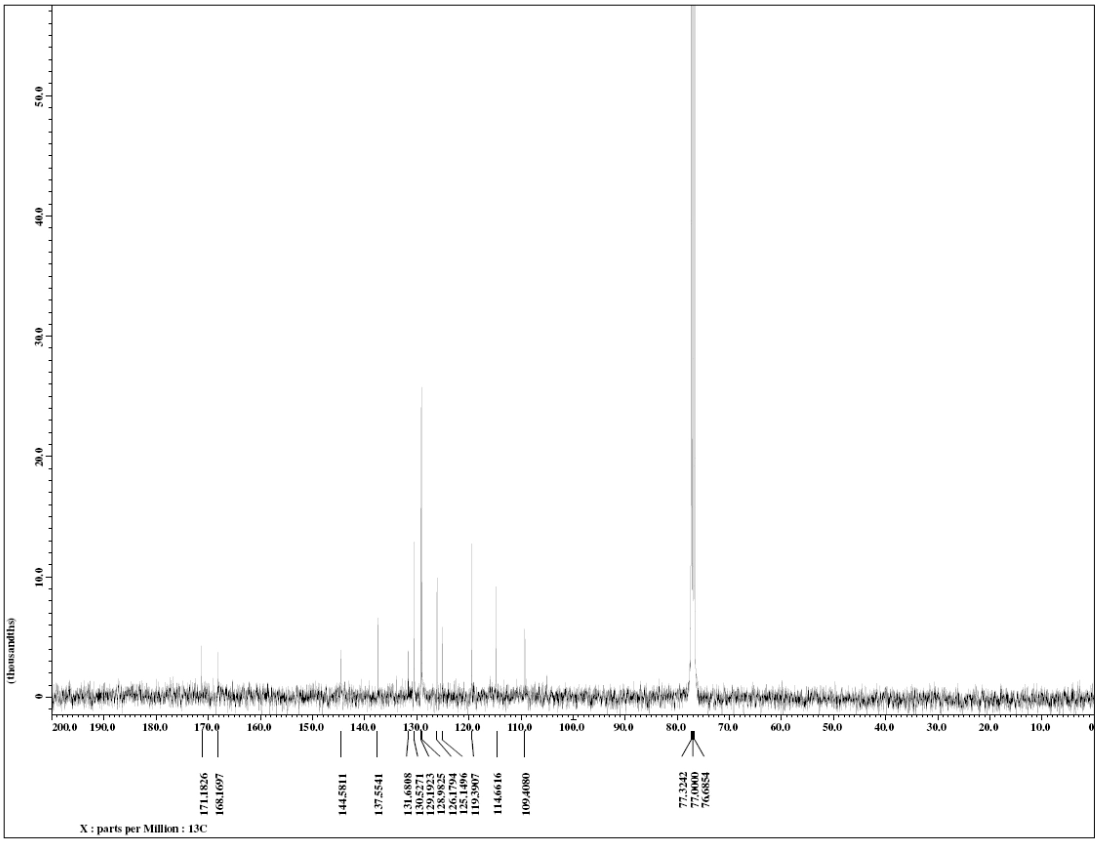
(thousandths)
171.1826
168.1697
144.5811
137.5541
131.6808
130.5271
129.1923
128.9825
126.1794
125.1496
119.3907
114.6616
109.4080
77.3342
77.0000
76.6854
X : parts per Million : 13C

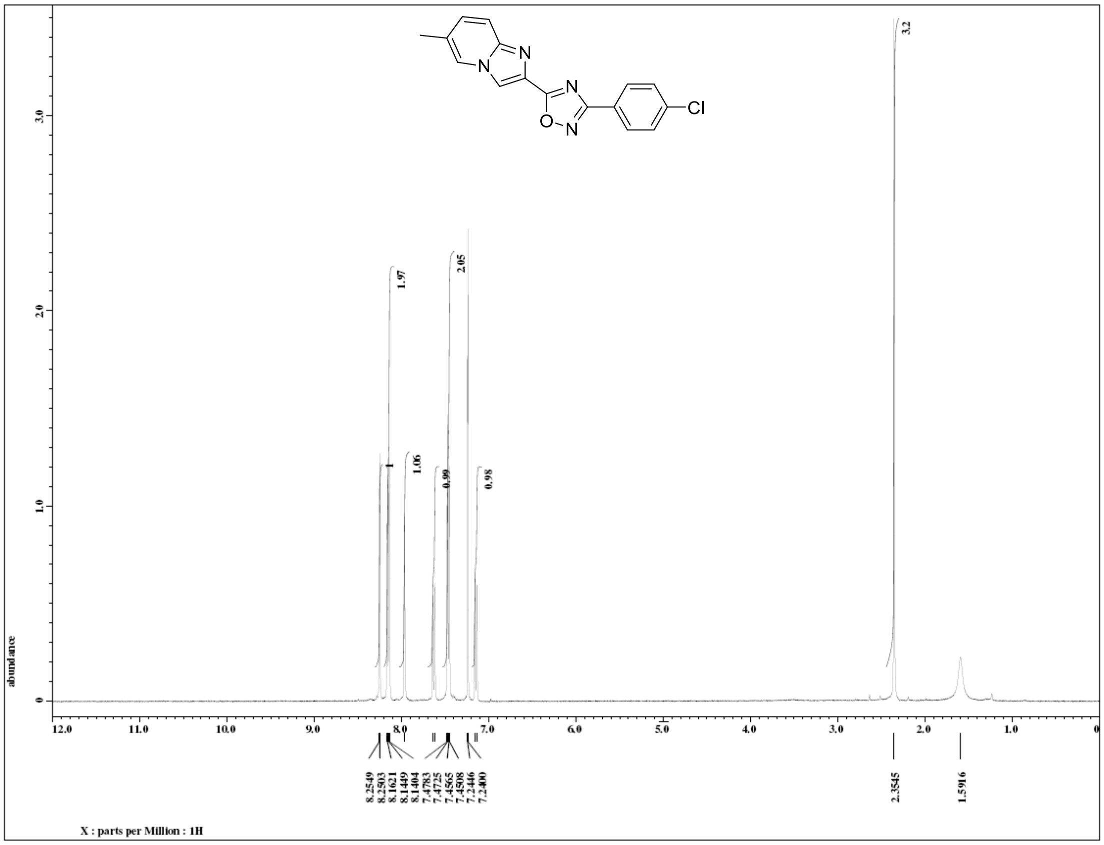

Cl
N
O
abundance
X : parts per Million : 1H

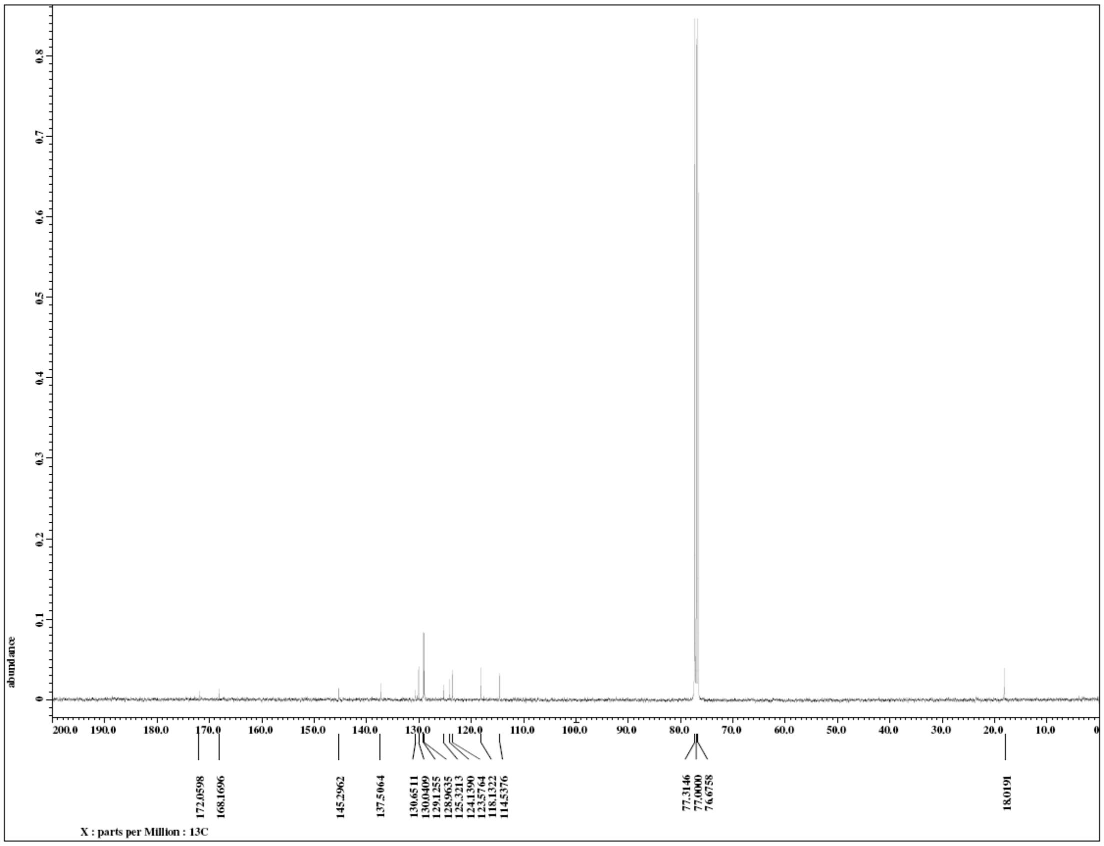

abundance
X : parts per Million : 13C

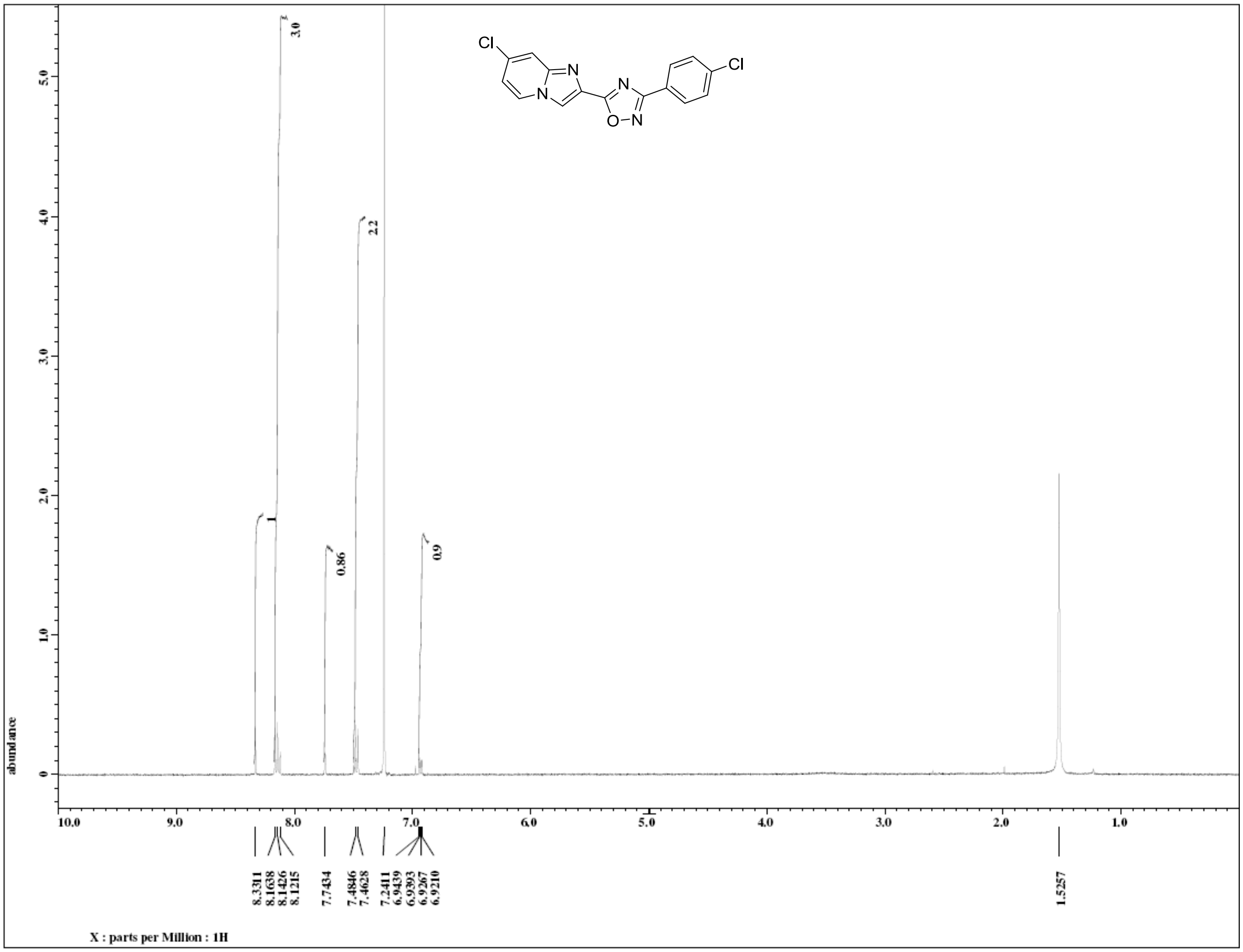
Cl
N
N
N
O
N
Cl
abundance
5.0
4.0
3.0
2.0
1.0
0
3.0
2.2
1
0.86
0.9
10.0
9.0
8.0
7.0
6.0
5.0
4.0
3.0
2.0
1.0
8.3311
8.1638
8.1426
8.1215
7.7434
7.4846
7.4628
7.2411
6.9439
6.9393
6.9267
6.9210
1.5257
X : parts per Million : 1H

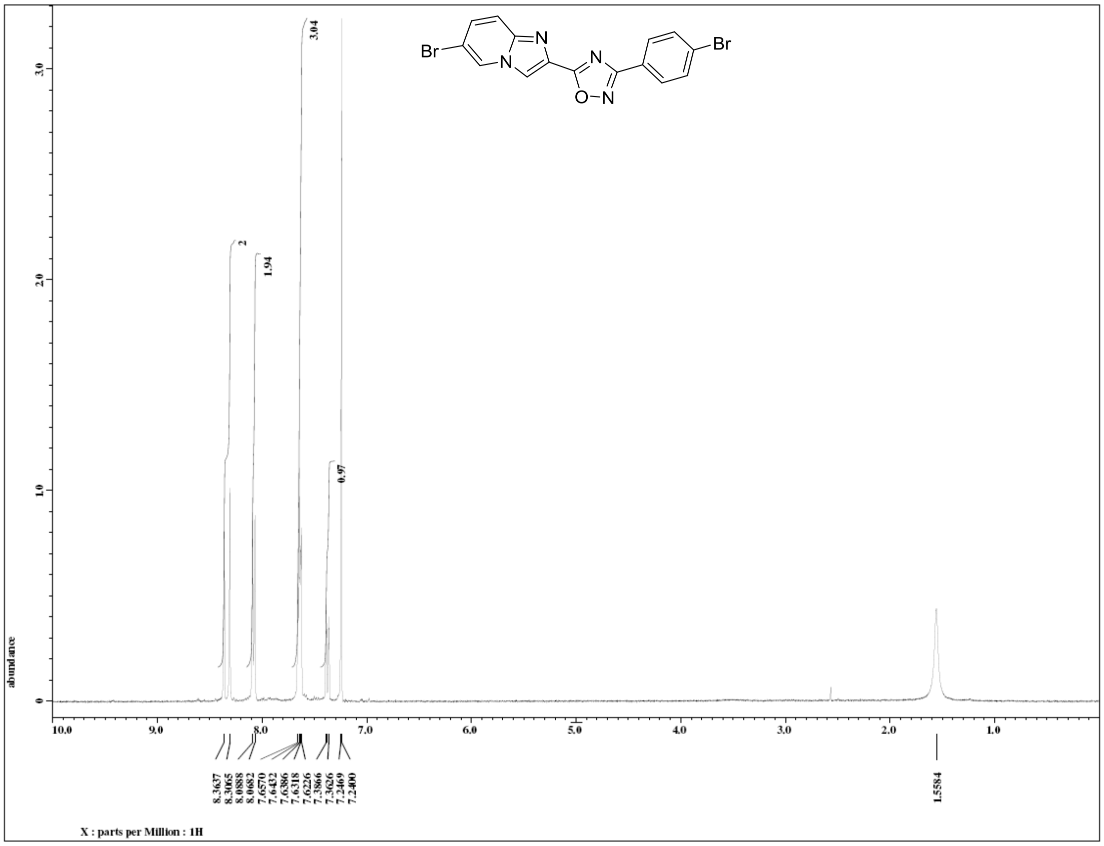
Br
N
N
O–N
N
Br
3.04
2
1.94
0.97
abundance
8.3637
8.3065
8.0888
8.0682
7.6570
7.6432
7.6386
7.6318
7.6226
7.3866
7.3626
7.2469
7.2400
1.5584
X : parts per Million : 1H

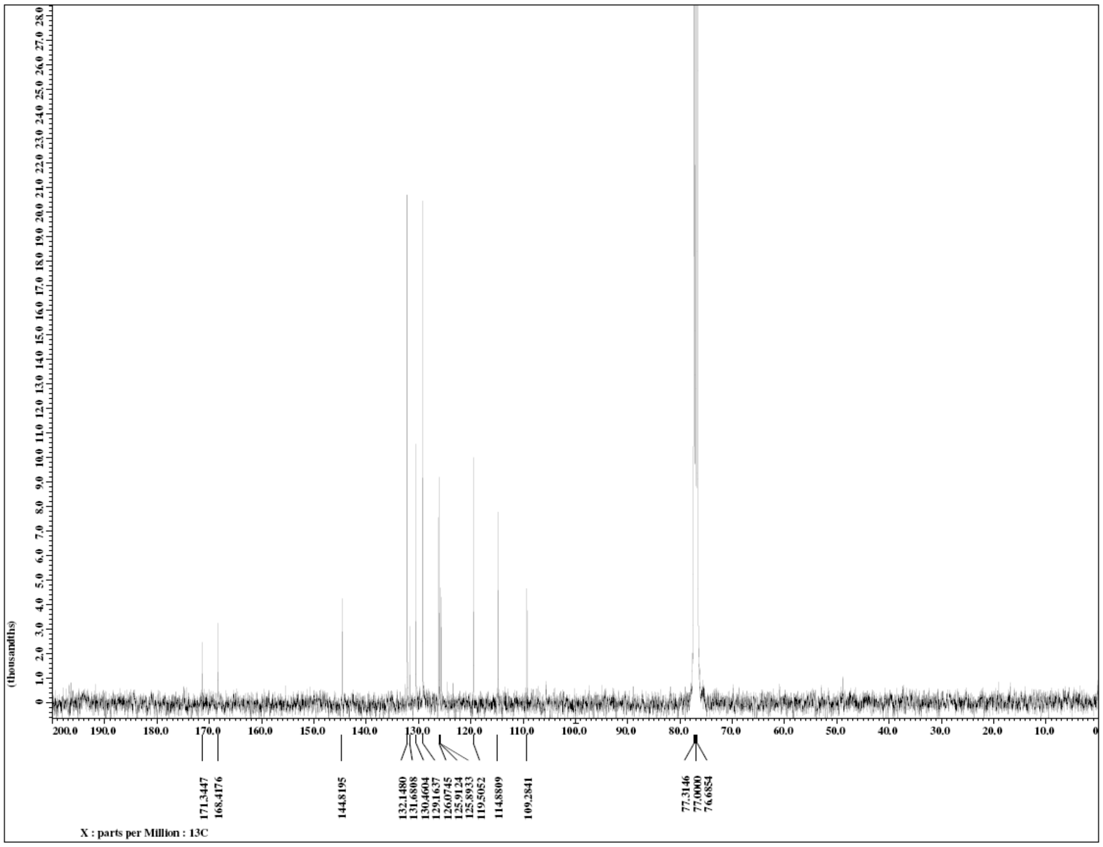
(thousandths)
171.3447
168.4176
144.8195
132.1480
131.6808
130.4604
129.1637
126.0745
125.9124
125.8933
119.5052
114.8809
109.2841
77.3146
77.0000
76.6854
X : parts per Million : 13C

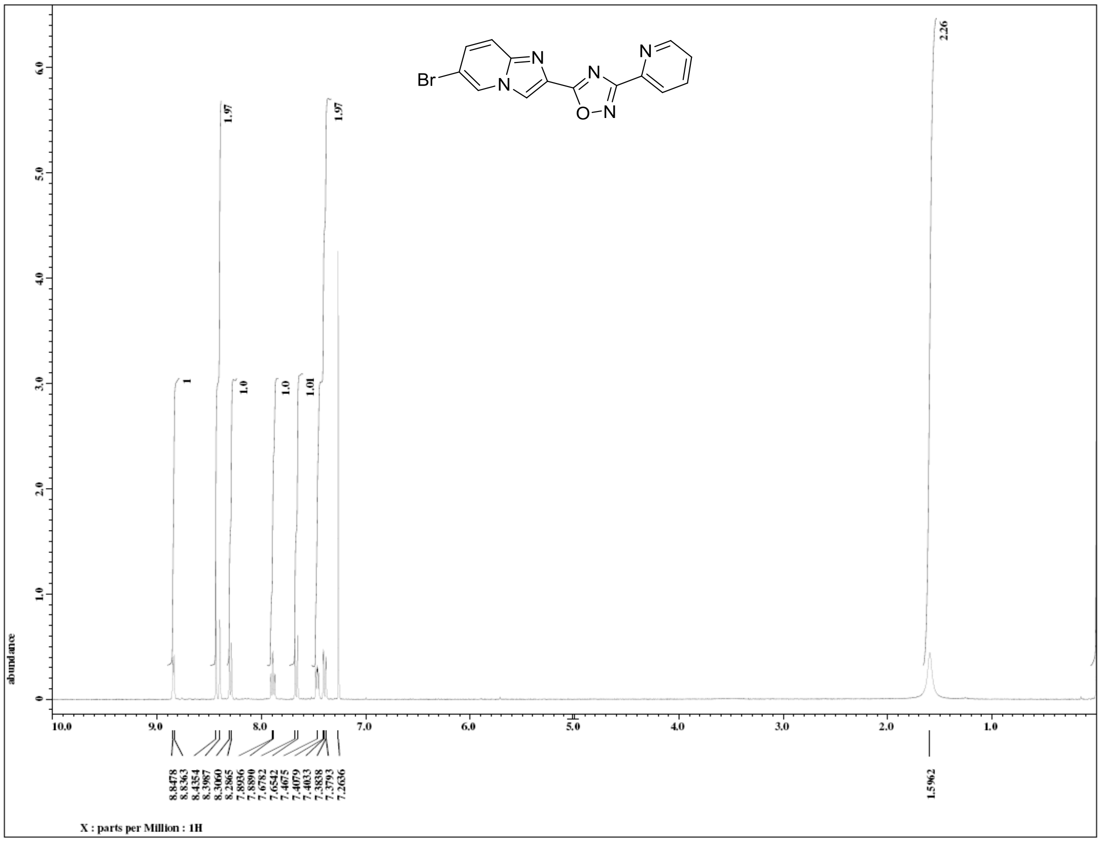
Br
N
N
N
O–N
N
abundance
X : parts per Million : 1H
8.8478
8.8363
8.4354
8.3987
8.3060
8.2865
7.8936
7.8890
7.6782
7.6542
7.4675
7.4079
7.4033
7.3838
7.3793
7.2636
1.5962
1
1.97
1.0
1.0
1.01
1.97
2.26

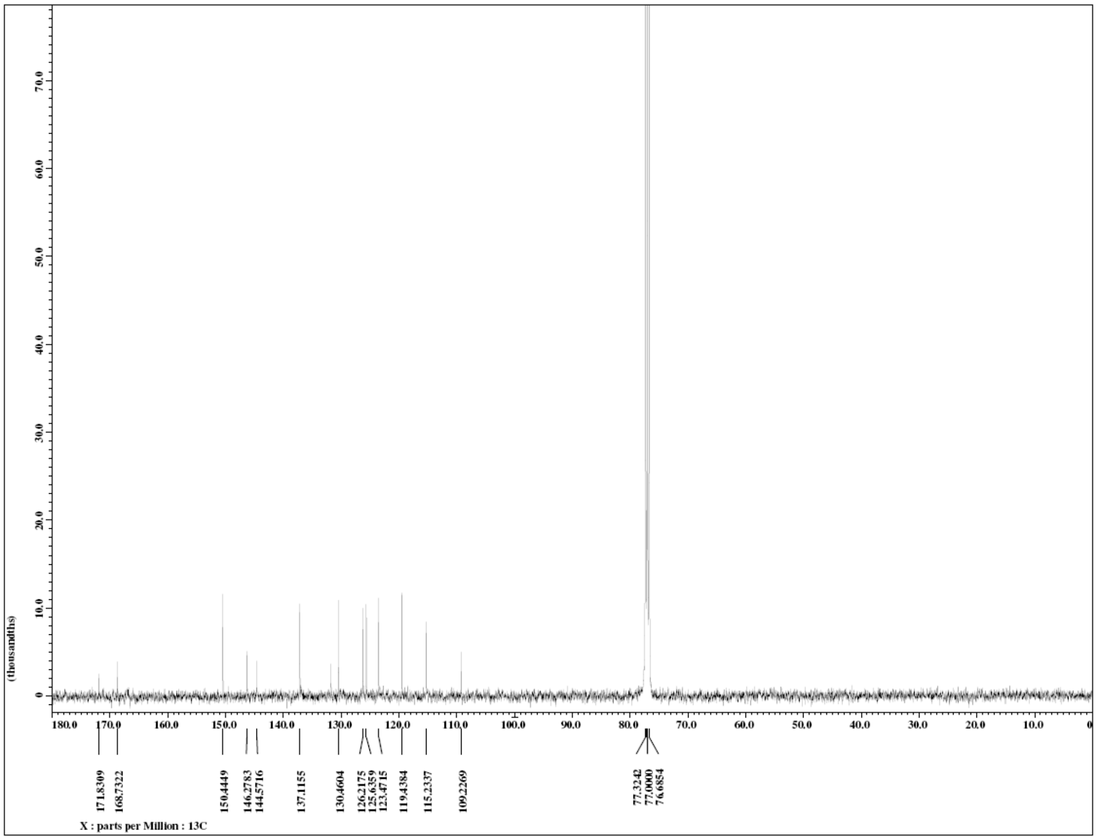
(thousandths)
X : parts per Million : 13C
171.8309
168.7322
150.4449
146.2783
144.5716
137.1155
130.4604
126.2175
125.6359
123.4715
119.4384
115.2337
109.2269
77.3242
77.0000
76.6854

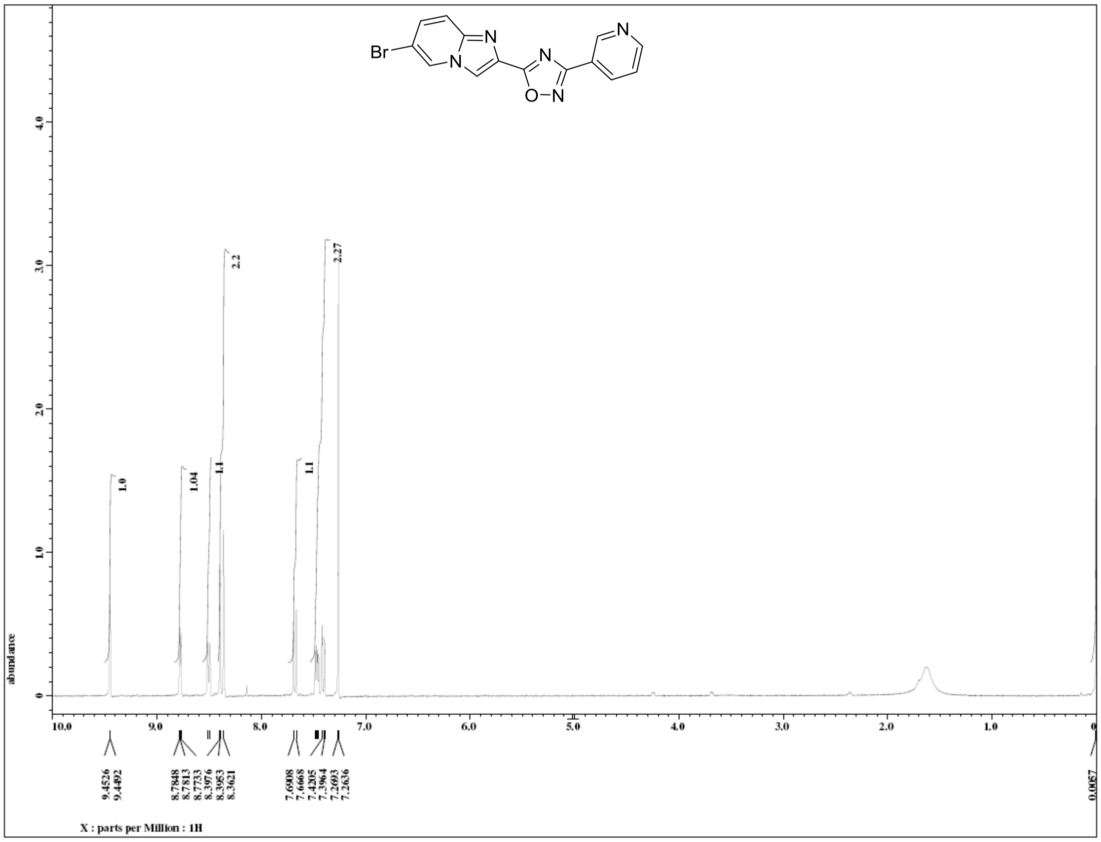
Br
N
N
N
O–N
N
abundance
X : parts per Million : 1H

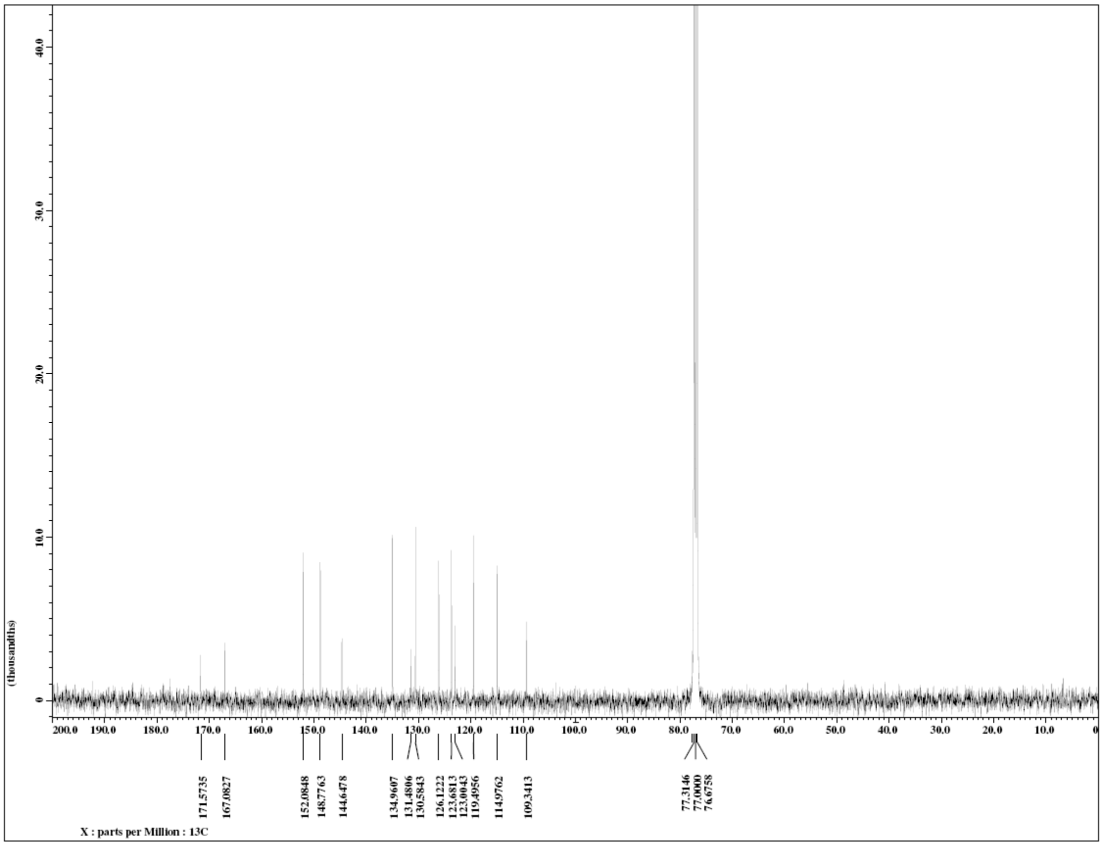
(thousandths)
X : parts per Million : 13C

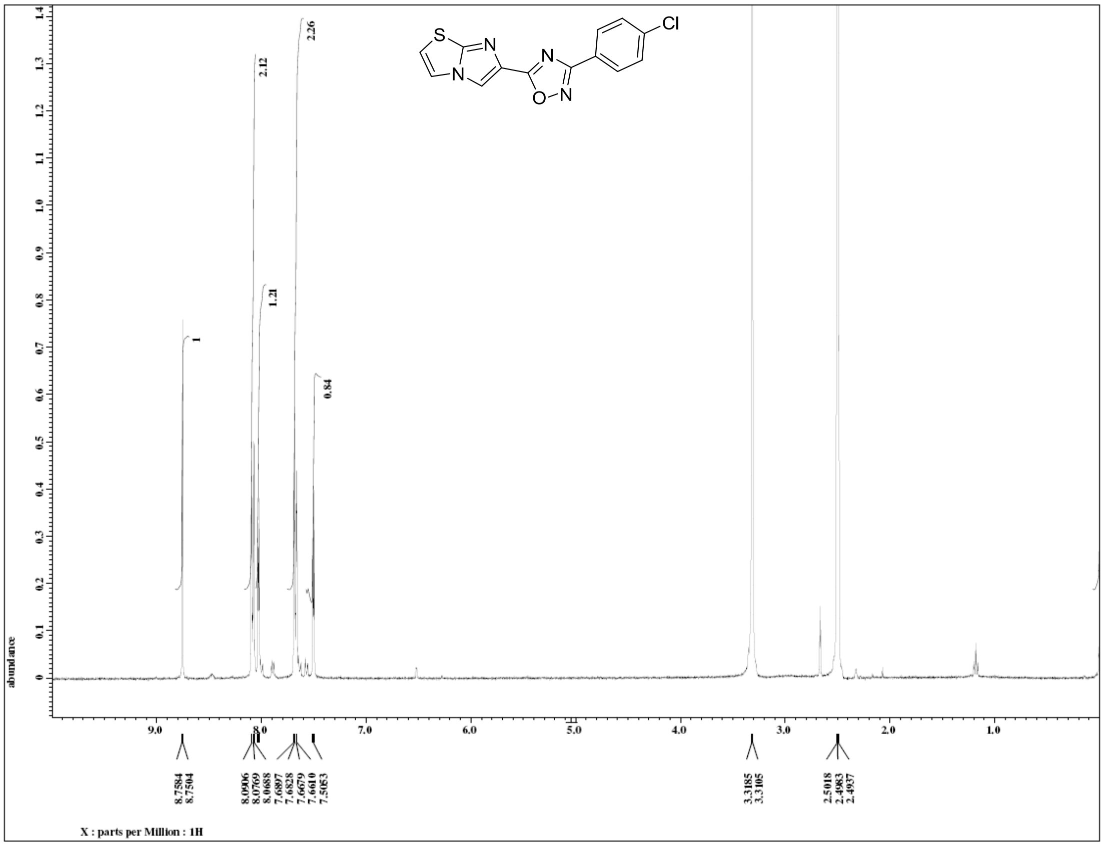
S
N
N
N
O–N
Cl
abundance
X : parts per Million : 1H

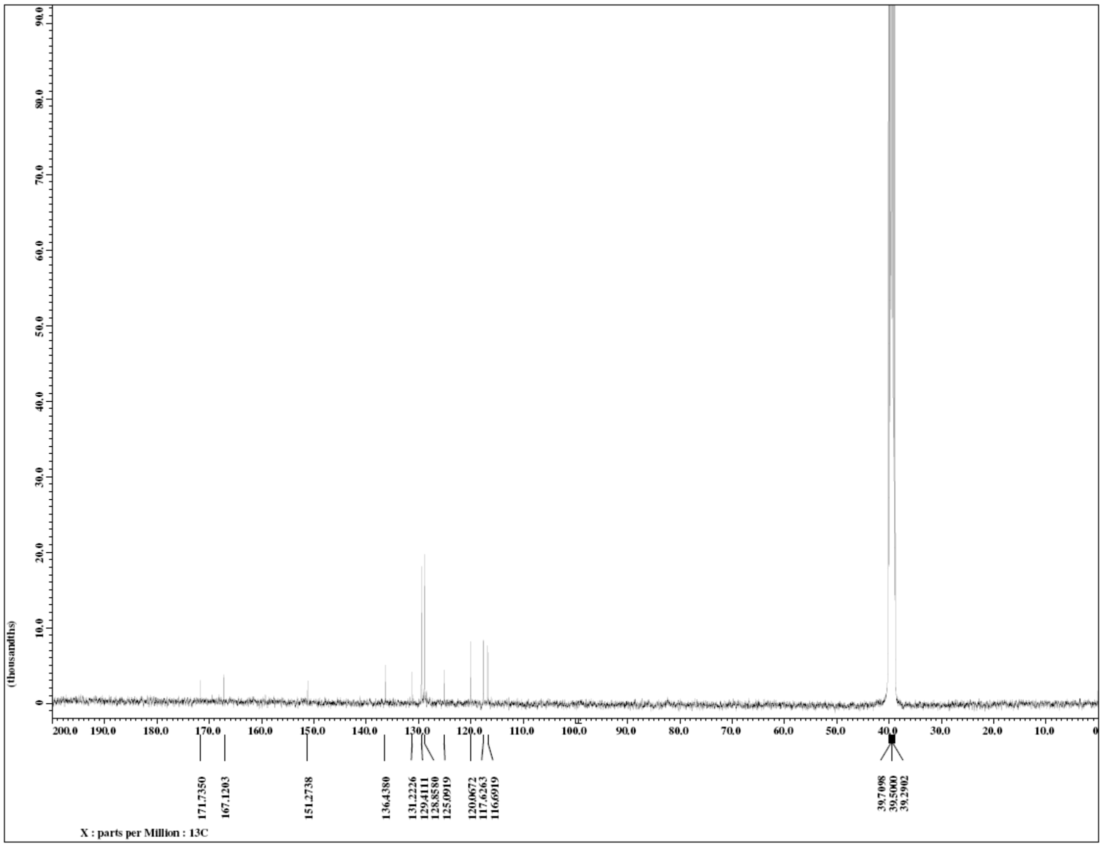
(thousandths)
X : parts per Million : 13C

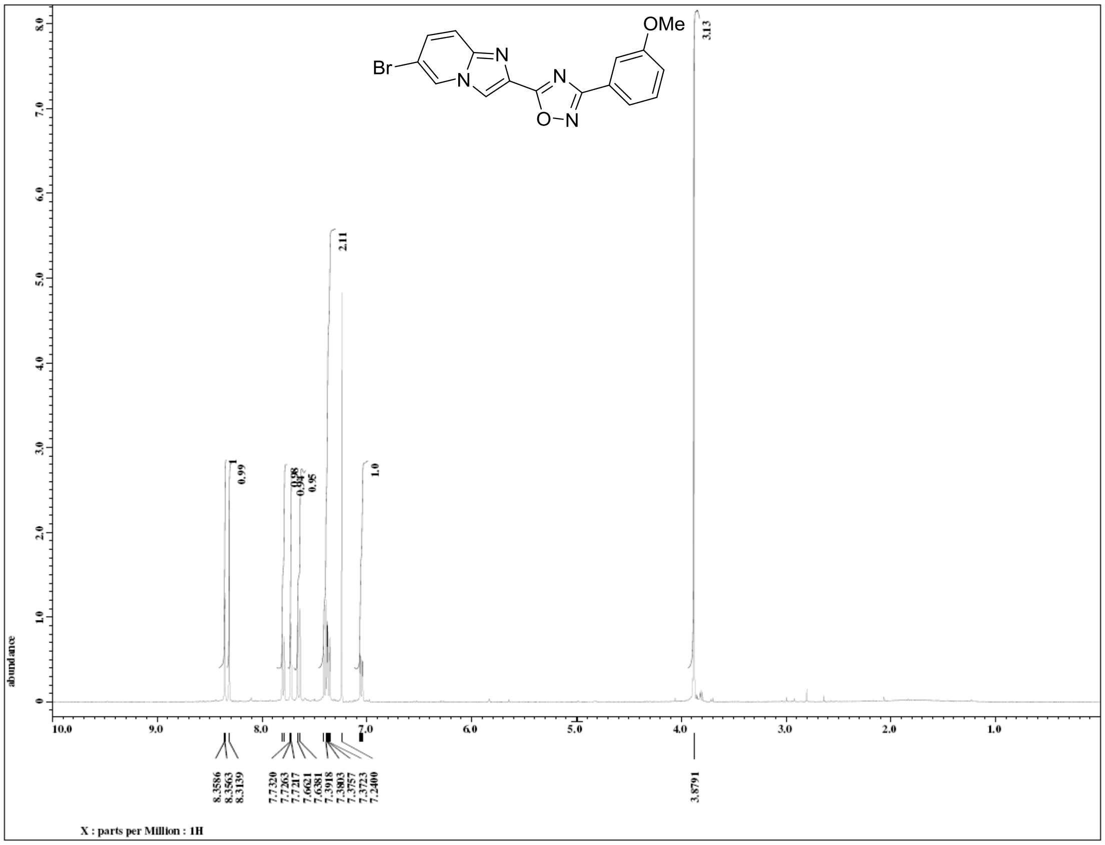
OMe
Br
N
N
O-N
abundance
8.3586
8.3563
8.3139
7.7320
7.7263
7.7217
7.6621
7.6381
7.3918
7.3803
7.3757
7.3723
7.2400
3.8791
X : parts per Million : 1H

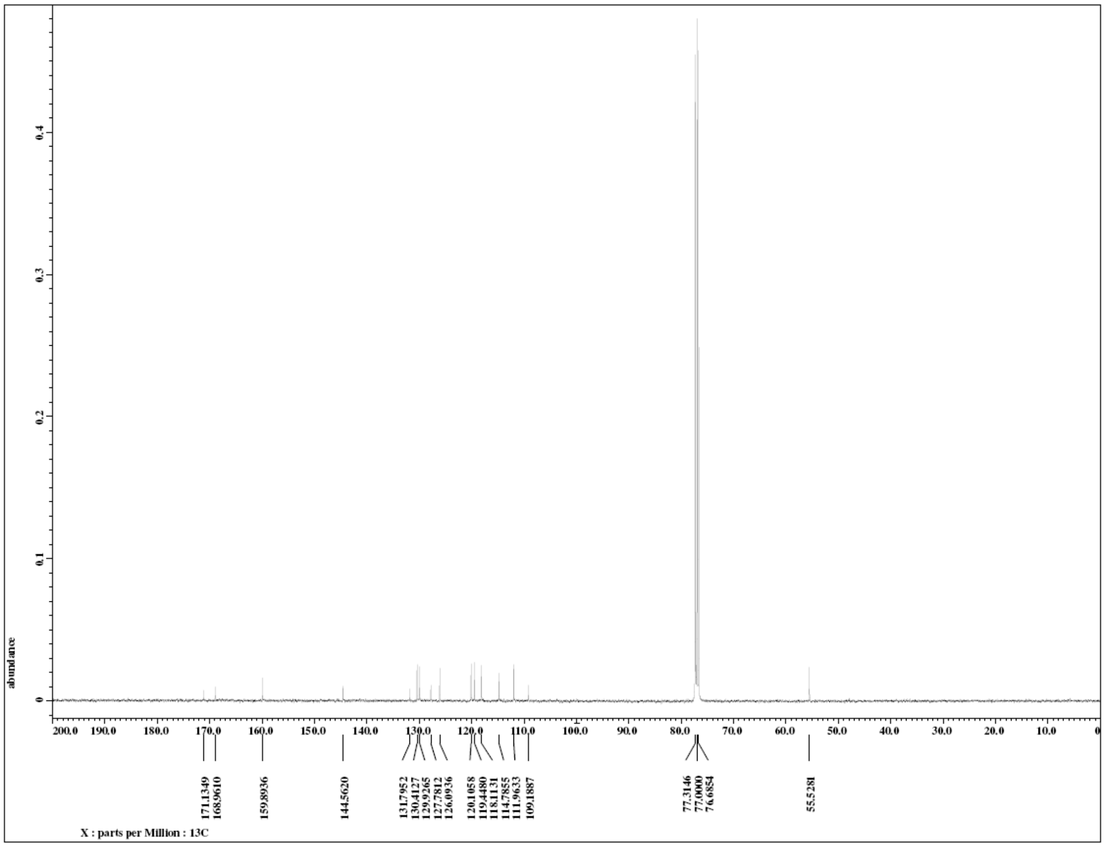
abundance
171.1349
168.9610
159.8936
144.5620
131.7952
130.4127
129.9265
127.7812
126.0936
120.1058
119.4480
118.1131
114.7855
111.9633
109.1887
77.3146
77.0000
76.6854
55.5281
X : parts per Million : 13C

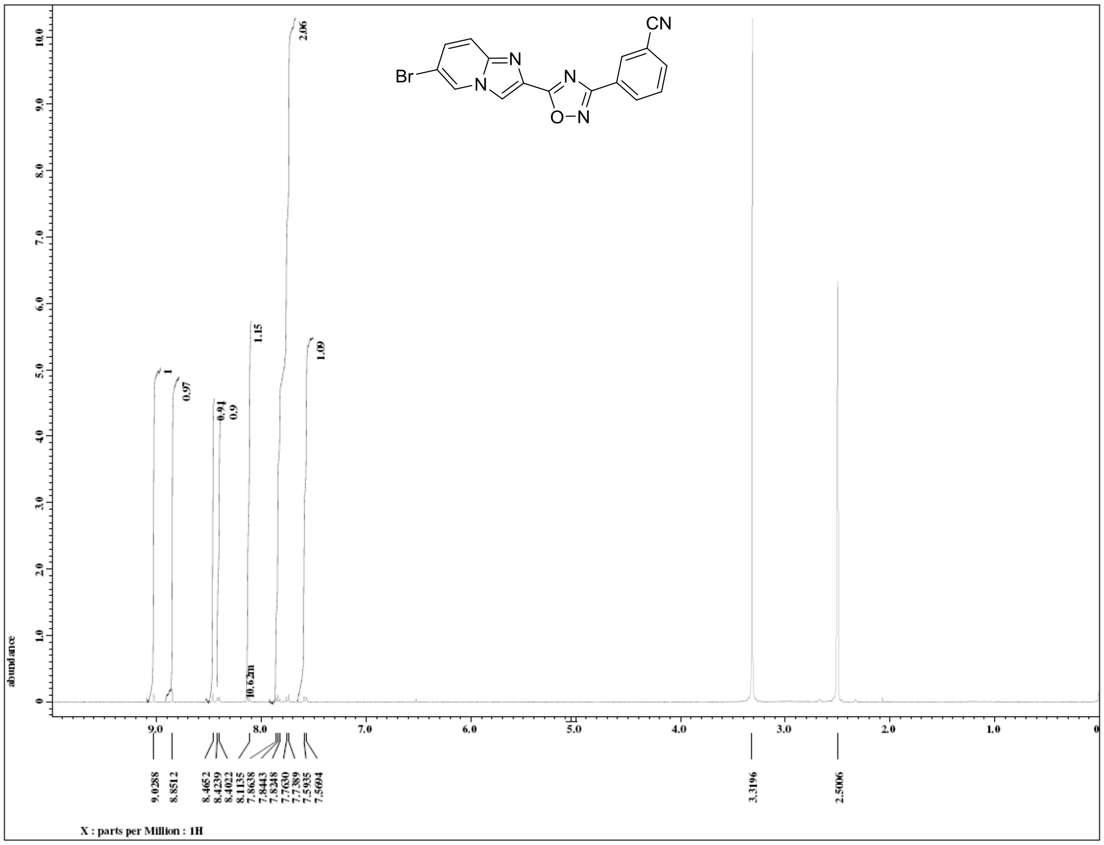
CN
Br
N
O-N
abundance
X : parts per Million : 1H
9.0288
8.8512
8.4652
8.4239
8.4022
8.1135
7.8638
7.8443
7.8248
7.7630
7.7389
7.5935
7.5694
3.3196
2.5006

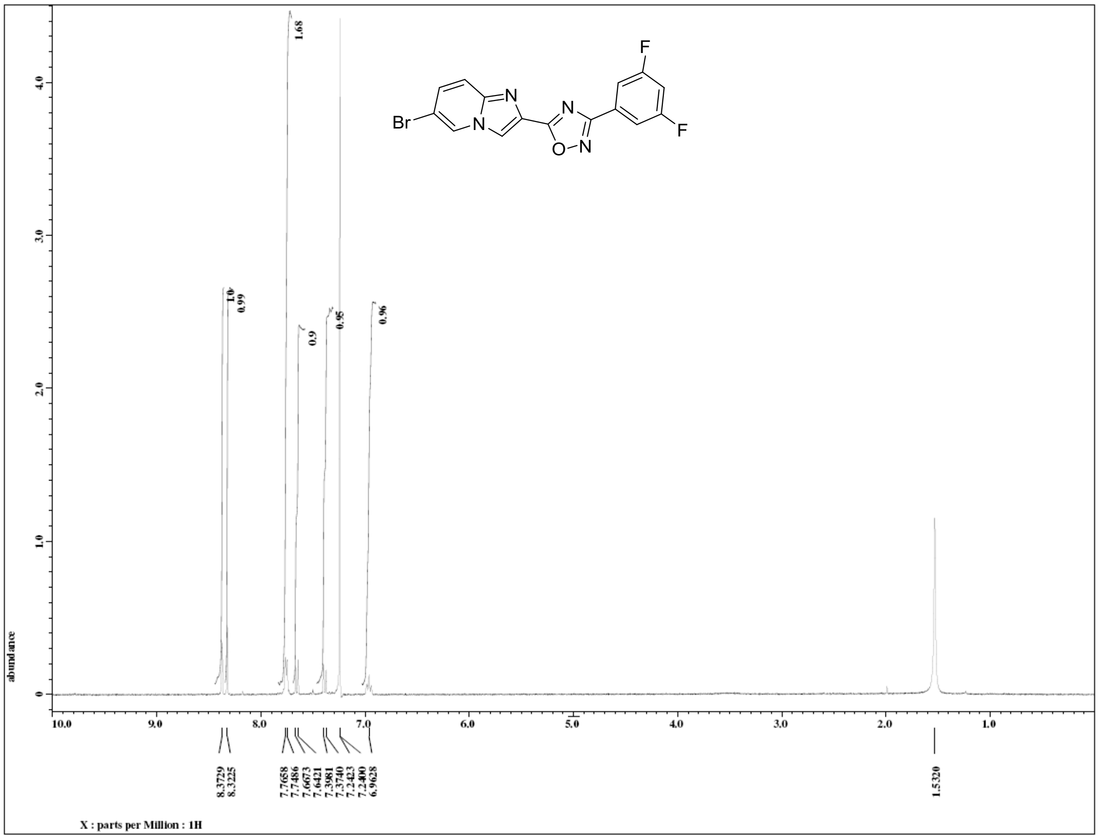

Br
N
N
O-N
F
F
1.68
1.0
0.99
0.9
0.95
0.96
abundance
8.3729
8.3225
7.7658
7.7486
7.6673
7.6421
7.3981
7.3740
7.2423
7.2400
6.9628
1.5320
X : parts per Million : 1H

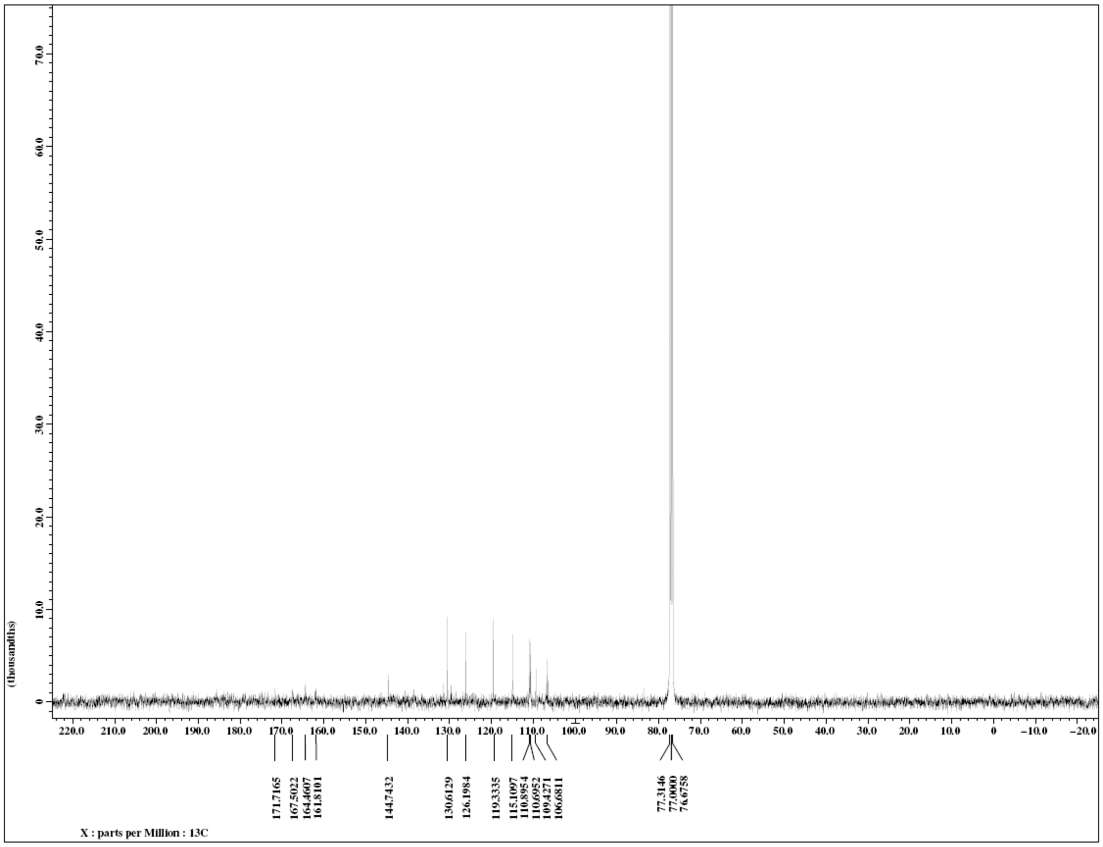

(thousandths)
171.7165
167.5022
164.4607
161.8101
144.7432
130.6129
126.1984
119.3335
115.1097
110.8954
110.6952
109.4271
106.6811
77.3146
77.0000
76.6758
X : parts per Million : 13C

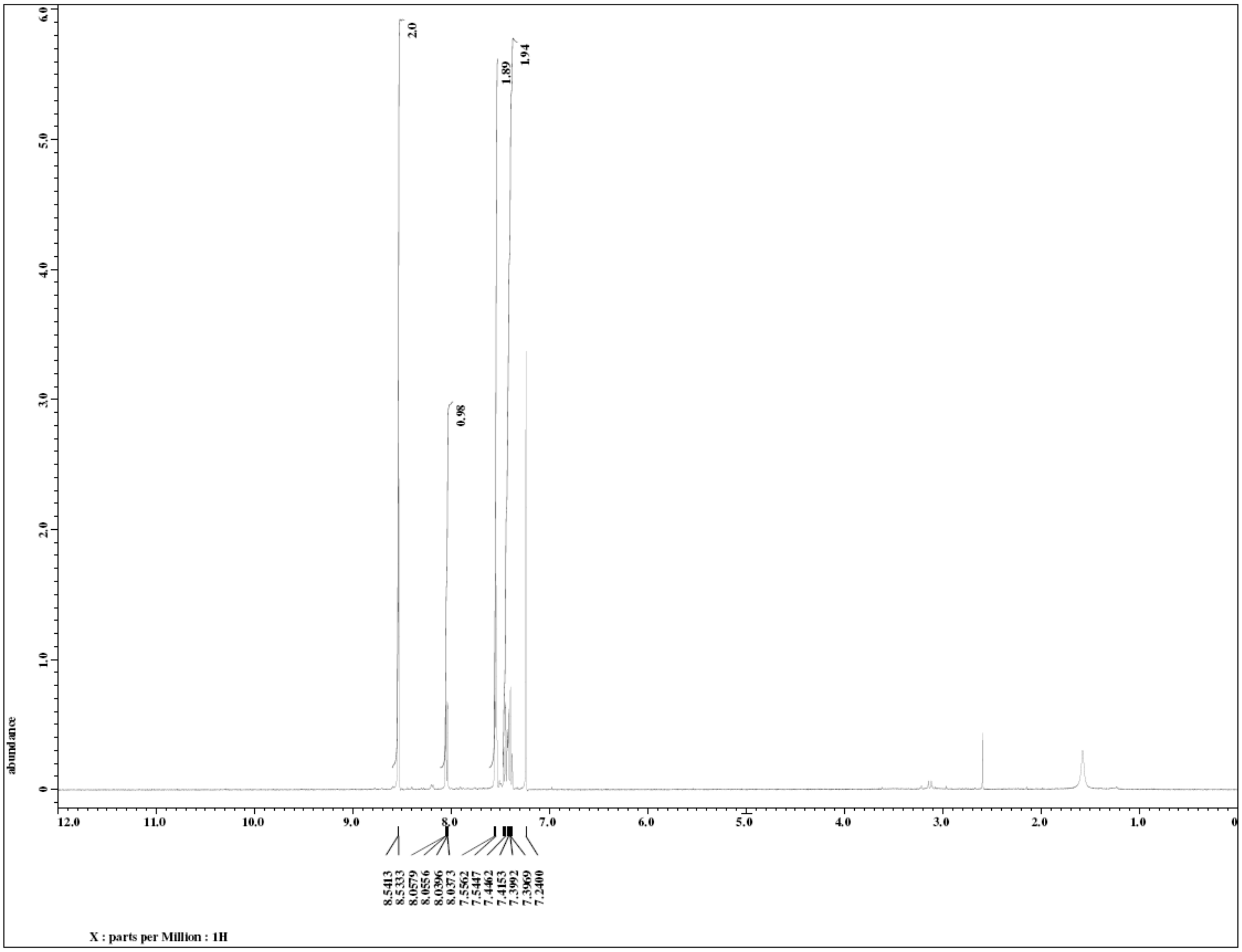

abundance
6.0
5.0
4.0
3.0
2.0
1.0
0
2.0
0.98
1.89
1.94
12.0
11.0
10.0
9.0
8.0
7.0
6.0
5.0
4.0
3.0
2.0
1.0
8.5413
8.5333
8.0579
8.0556
8.0396
8.0373
7.5562
7.5447
7.4462
7.4153
7.3992
7.3969
7.2400
X : parts per Million : 1H

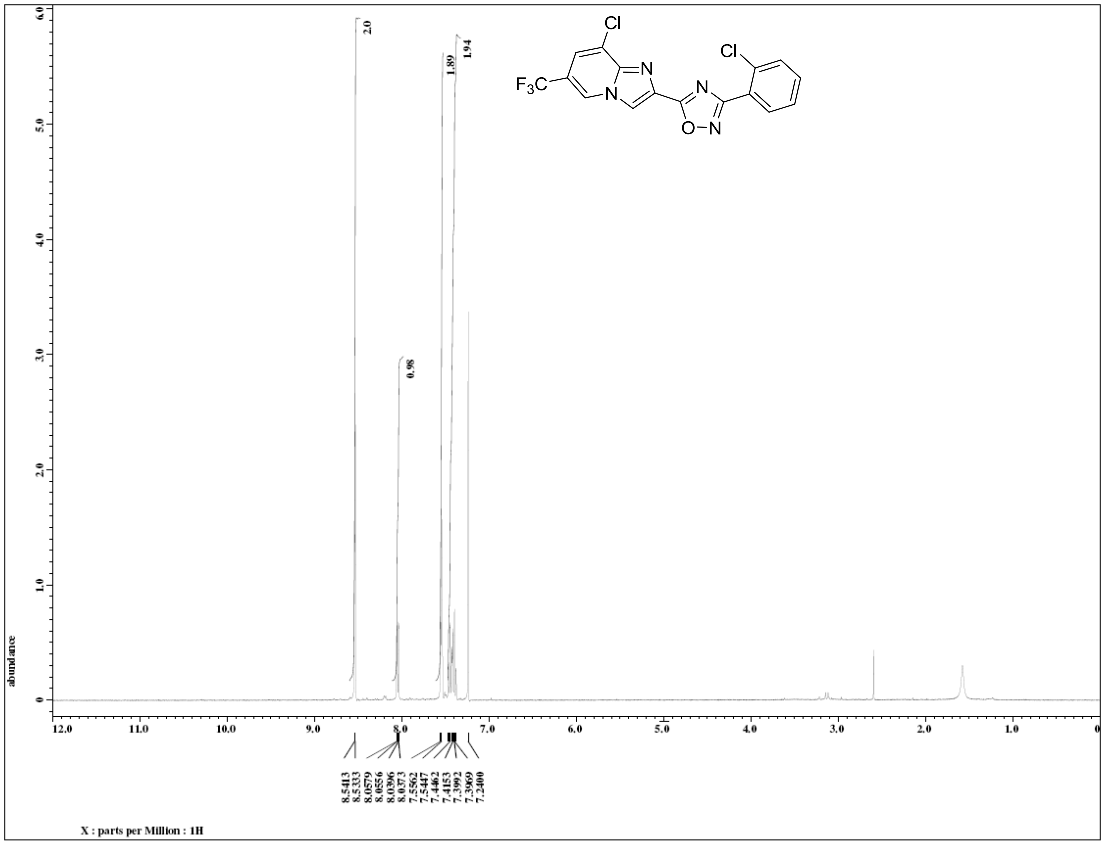
Cl
F3C
N
N
N
O–N
Cl
abundance
2.0
0.98
1.89
1.94
8.5413
8.5333
8.0579
8.0556
8.0396
8.0373
7.5562
7.5447
7.4462
7.4153
7.3992
7.3969
7.2400
X : parts per Million : 1H

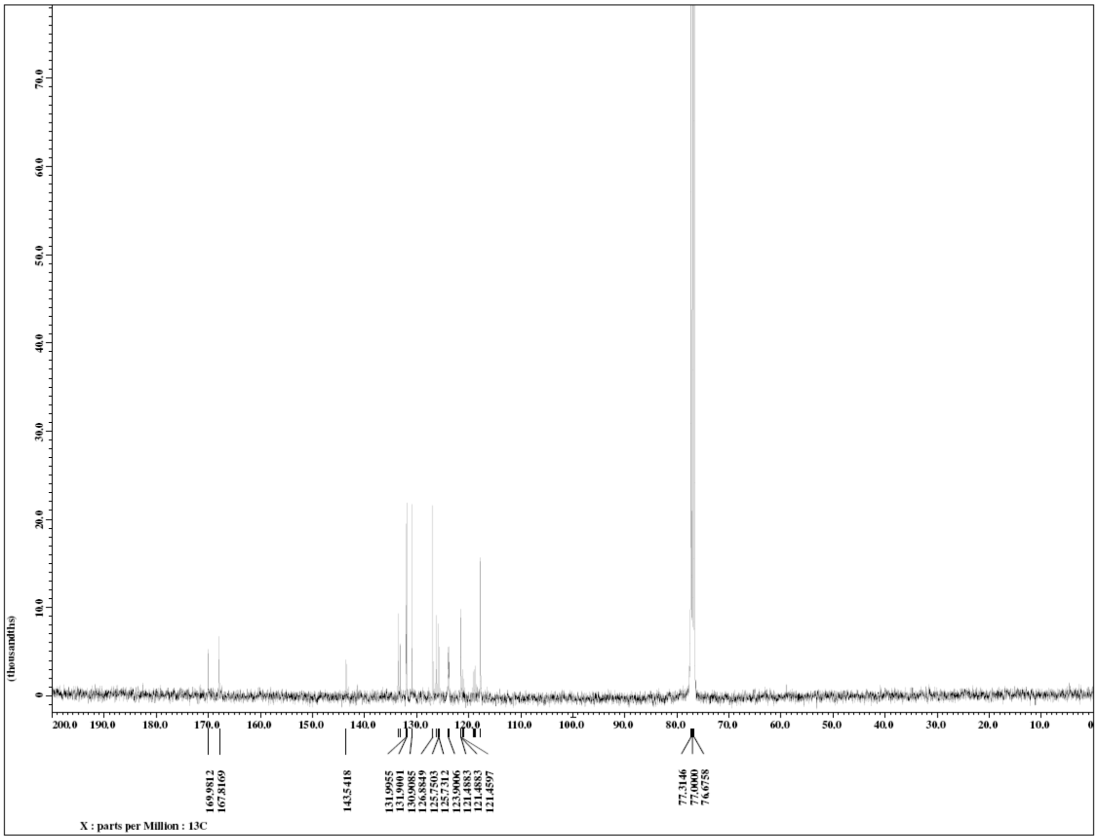
(thousandths)
169.9812
167.8169
143.5418
131.9955
131.9001
130.9085
126.8849
125.7503
125.7312
123.9006
121.4883
121.4883
121.4597
77.3146
77.0000
76.6758
X : parts per Million : 13C

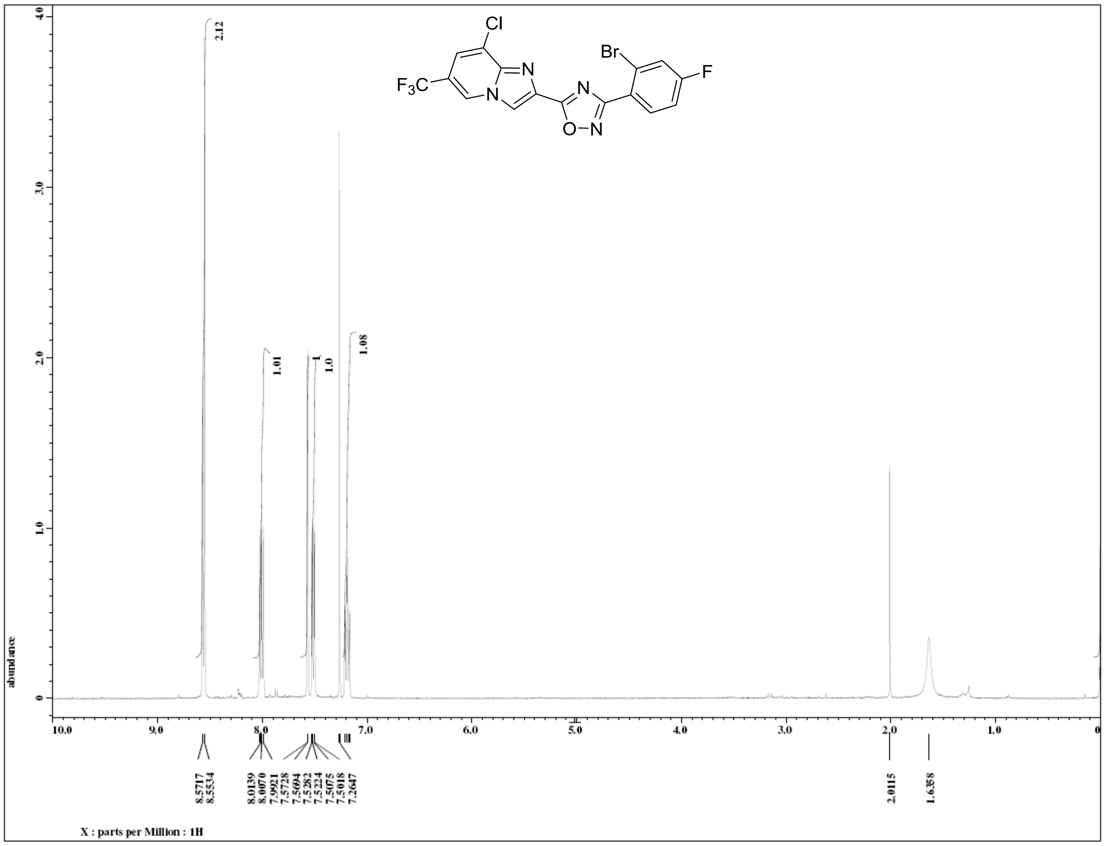

Cl
Br
F
F_3C
N
N
N
O–N
abundance
X : parts per Million : 1H
2.12
1.01
1.0
1.08
8.5717
8.5534
8.0139
8.0070
7.9921
7.5728
7.5694
7.5282
7.5224
7.5075
7.5018
7.2647
2.0115
1.6358

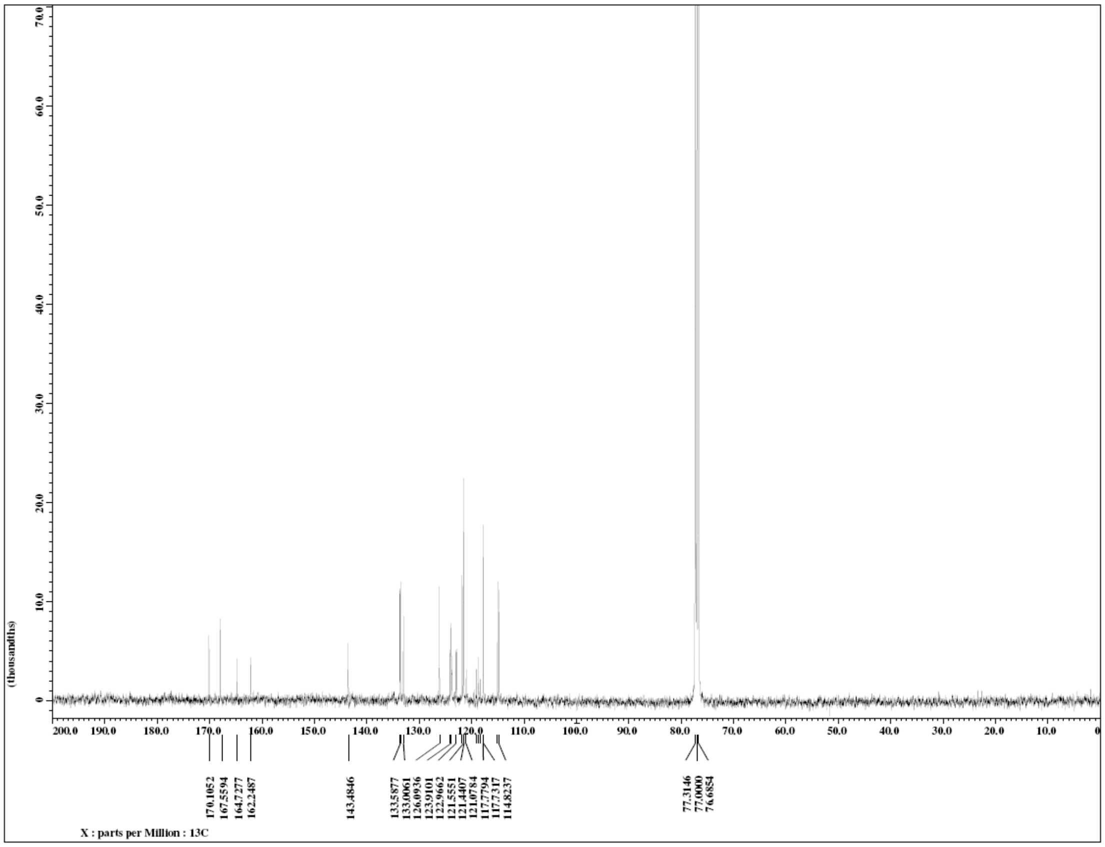

(thousandths)
X : parts per Million : 13C
170.1052
167.5594
164.7277
162.2487
143.4846
133.5877
133.0061
126.0936
123.9101
122.9662
121.5551
121.4407
121.0784
117.7794
117.7317
114.8237
77.3146
77.0000
76.6854
